# Accelerated hypometabolism with disease progression associated with faster cognitive decline among amyloid positive patients

**DOI:** 10.3389/fnins.2023.1151820

**Published:** 2023-04-14

**Authors:** Zhengshi Yang, Jeffrey L. Cummings, Jefferson W. Kinney, Dietmar Cordes, the Alzheimer’s Disease Neuroimaging Initiative

**Affiliations:** ^1^Cleveland Clinic Lou Ruvo Center for Brain Health, Las Vegas, NV, United States; ^2^Department of Brain Health, University of Nevada, Las Vegas, Las Vegas, NV, United States; ^3^Chambers-Grundy Center for Transformative Neuroscience, Pam Quirk Brain Health and Biomarker Laboratory, Department of Brain Health, School of Integrated Health Sciences, University of Nevada, Las Vegas, Las Vegas, NV, United States; ^4^Department of Psychology and Neuroscience, University of Colorado Boulder, Boulder, CO, United States

**Keywords:** FDG-PET, Alzheimer’s continuum, brain metabolism, cognitive decline, AD

## Abstract

**Objective:**

To evaluate the progression of brain glucose metabolism among participants with biological signature of Alzheimer’s disease (AD) and its relevance to cognitive decline.

**Method:**

We studied 602 amyloid positive individuals who underwent 18F-fluorodeoxyglucose PET (FDG-PET) scan, 18F-AV-45 amyloid PET (AV45-PET) scan, structural MRI scan and neuropsychological examination, including 116 cognitively normal (CN) participants, 314 participants diagnosed as mild cognitive impairment (MCI), and 172 participants diagnosed as AD dementia. The first FDG-PET scan satisfying the inclusion criteria was considered as the baseline scan. Cross-sectional analysis were conducted with the baseline FDG-PET data to compare the regional differences between diagnostic groups after adjusting confounding factors. Among these participants, 229 participants (55 CN, 139 MCI, and 35 AD dementia) had two-year follow-up FDG-PET data available. Regional glucose metabolism was computed and the progression rates of regional glucose metabolism were derived from longitudinal FDG-PET scans. Then the group differences of regional progression rates were examined to assess whether glucose metabolism deficit accelerates or becomes stable with disease progression. The association of cognitive decline rate with baseline regional glucose metabolism, and progression rate in longitudinal data, were evaluated.

**Results:**

Participants with AD dementia showed substantial glucose metabolism deficit than CN and MCI at left hippocampus, in addition to the traditionally reported frontal and parietal–temporal lobe. More substantial metabolic change was observed with the contrast AD – MCI than the contrast MCI – CN, even after adjusting time duration since cognitive symptom onset. With the longitudinal data, glucose metabolism was observed to decline the most rapidly in the AD dementia group and at a slower rate in MCI. Lower regional glucose metabolism was correlated to faster cognitive decline rate with mild–moderate correlations, and the progression rate was correlated to cognitive decline rate with moderate-large correlations.

**Discussion and conclusion:**

Hippocampus was identified to experience hypometabolism in AD pathology. Hypometabolism accelerates with disease progression toward AD dementia. FDG-PET, particularly longitudinal scans, could potentially help predict how fast cognition declines and assess the impact of treatment in interventional trials.

## Introduction

Alzheimer’s dementia was traditionally diagnosed based on the clinical syndrome, with the confirmation of the clinical diagnose requiring autopsy (McKhann et al., 1984). In the last decades, the field has shifted from the syndromal definition toward the biological definition of AD and various biomarkers have been developed to support the diagnosis of AD in living patients. Among these biomarkers, abnormal amyloid deposition was recognized as the defining signature of AD (Jack et al., 2018). A positive amyloid positron emission tomography (PET) scan, regardless of tau pathology and neurodegeneration, indicates the participant has AD pathologic change, distinguishing those on the AD continuum from the ones with similar phenotypes but without underlying biological AD pathology. Reduction of brain glucose metabolism, assessed by 18F-fluorodeoxyglucose PET (FDG-PET) imaging, was recognized as a biomarker to characterize neurodegeneration in AD (Mosconi et al., 2008b; Shi et al., 2009; Yuan et al., 2009). The biological differences between the molecular imaging with amyloid PET and metabolic imaging with FDG-PET allows these measures to provide important complementary information. The importance and role of both measures was recognized in the National Institute on Aging and Alzheimer’s Association (NIA-AA) research framework (Jack et al., 2018).

Both hypometabolism and hippocampal atrophy, assessed by structural magnetic resonance imaging (MRI), were acknowledged to be the biomarkers of neurodegeneration in AD, however, FDG-PET was demonstrated to explain the biological changes independent of hippocampal atrophy (Ou et al., 2019). The discrepancy of the biological mechanisms behind structural MRI and FDG-PET could explain their relevant but differentiated roles in AD research. FDG-PET was used to characterize the pathology in AD prior to the emergence of amyloid PET, thus the participants in the early studies were not biologically confirmed to be on the AD continuum. These studies provided valuable insights in understanding the metabolic changes evidenced in AD (Herholz et al., 2002; Bohnen et al., 2012; Rice and Bisdas, 2017), with the drawback that the unknown amyloid status could increase participant heterogeneity and thus undermine the capability of FDG-PET in unveiling the pathological changes in AD. In fact, while hippocampal hypometabolism was observed in very few cognitively normal elderly who had post-mortem diagnosis of definite AD (Mosconi et al., 2009), participants with clinically diagnosed AD did not show significant metabolic deficit compared to cognitively normal participants even with a large cohort (Herholz et al., 2002). Amyloid positivity as an inclusion criterion is an emerging trend in AD interventional trials and observational research; refining the FDG-PET analysis on a large cohort of participants with stratified amyloid status is needed for better capturing AD-specific metabolic changes. These studies can allow better understanding of AD mechanisms and processes, and may represent an outcome measure helpful in evaluating the therapeutic effects of treatment in interventional trials.

Moreover, while hypometabolism was well recognized as one of the pathological changes in AD dementia, it remains to be determined whether hypometabolism progresses constantly, or slows down and reaches a plateau similar to Aβ deposition, or accelerates with disease progression. Synapse loss and neurodegeneration are the neuropathological changes most strongly correlated with clinical symptoms in AD and are associated with hypometabolism (Strom et al., 2021). A normal FDG-PET scan (no hypometabolism on visual read) at baseline could predict clinical stability with no or limited decline (Iaccarino et al., 2019), and the abnormalities on FDG-PET could predict progression from MCI to AD dementia (Chételat et al., 2003; Mosconi et al., 2004; Anchisi et al., 2005). These findings support the close relationship between glucose metabolism and cognitive decline, but much less attention is paid to the value of longitudinal FDG-PET scans in predicting cognitive decline. To function as an objective outcome measure in an interventional study, FDG-PET must be sensitive enough to detect subtle metabolic changes in a relatively short period. In addition, it is important to evaluate if the progression rate of glucose metabolism, besides the baseline glucose metabolism, is informative of cognitive decline rate, determining the prognostic value of FDG-PET in informing if the intervention could slow down further cognitive decline.

Therefore, we conducted comparisons of regional glucose metabolism among participants with confirmed positive amyloid PET scans at different disease stages, including normal cognition (CN), prodromal AD (MCI) and AD dementia. Group comparisons of the regional glucose metabolism among these diagnostic groups were carried out using baseline scans. For the participants with follow-up FDG-PET scans available, considering the three groups in our study represent different stages of AD, we examined the group differences of FDG-PET progression rate to unveil if hypometabolism accelerates over disease progression. In addition, it also clarifies if FDG-PET is able to detect metabolic changes in a short period. Correlation analysis between glucose metabolism and cognitive decline rate, quantified with the Clinical Dementia Rating – Sum of Boxes (CDR-SB) score, were carried out to assess the prognostic value of FDG-PET scan in predicting cognitive decline. We hypothesized that (1) hypometabolism progresses more rapidly at the advanced disease stage, and (2) lower glucose metabolism at baseline and faster metabolic decline are associated with more rapid cognitive decline.

Our goal was to better capture metabolic changes over disease progression among population with biologically-confirmed AD pathology, and to evaluate the association of cognitive decline rate with regional glucose metabolism and its progression rate, demonstrating the potential utility of (longitudinal) FDG-PET scans in assessing the therapeutic effect in interventional trials.

## Methods

### Subjects

**Figure 1:**
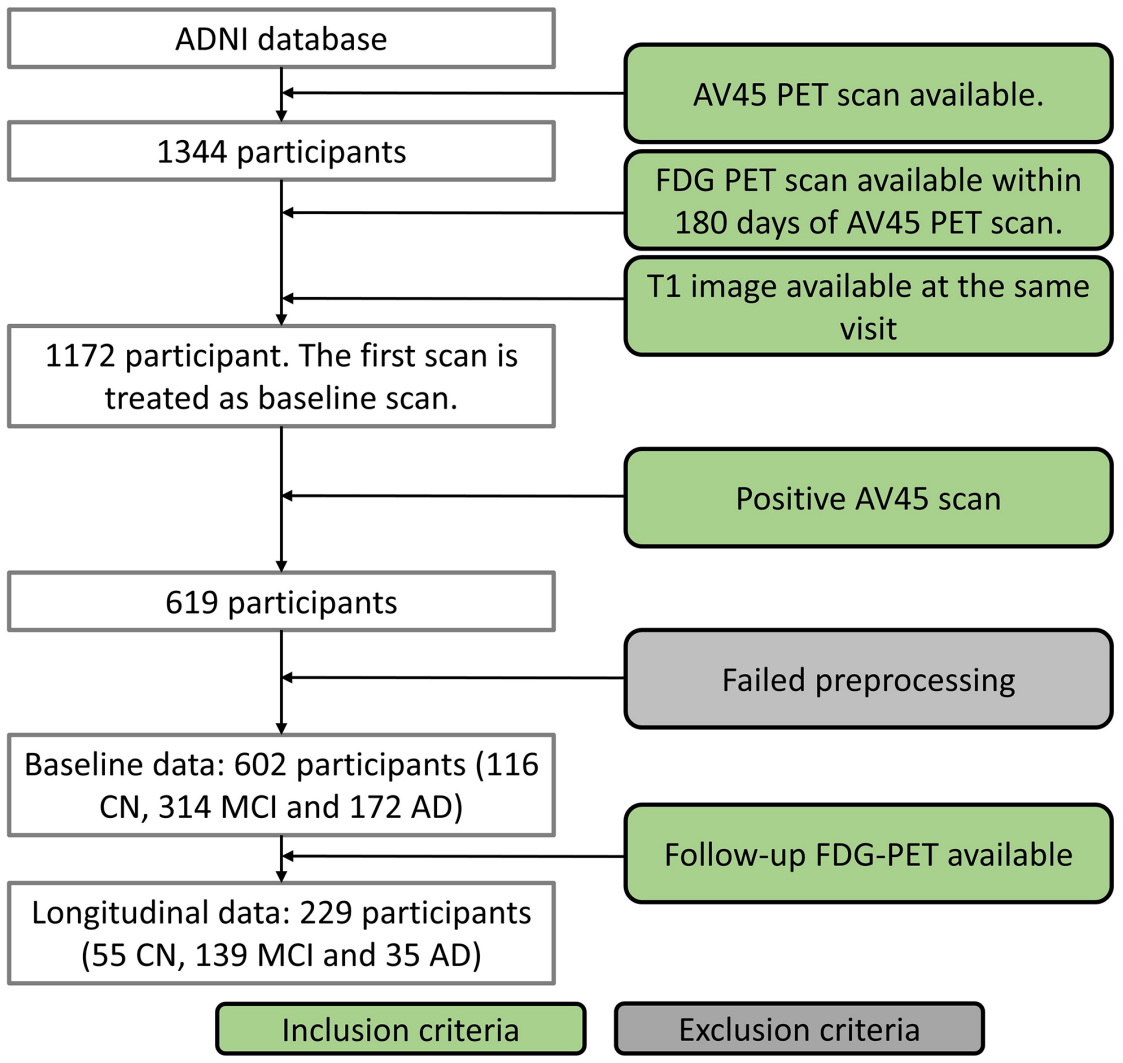
Participant inclusion flowchart.

Data used in this study were obtained from Alzheimer’s Disease Neuroimaging Initiative (ADNI)^1^ database in February 2022. The study was approved by each participating ADNI site’s local Institutional Review Board as documented on the ADNI website. All participants gave written, informed consent. The sponsors for ADNI are listed in the Acknowledgement. Of the whole ADNI cohort, 1,344 individuals were identified to have 18F-AV-45 (AV45) amyloid PET (AV45-PET) scans available. We excluded the subjects who were not under AD-track based on the brain amyloid status. The AV45-PET data were processed by following ADNI AV45-PET analysis pipeline (Landau et al., 2012). Briefly, the AV45-PET composite standardized uptake value ratio (SUVR) was averaged across extensive neocortical regions and then normalized with the whole cerebellum as the reference region. Participants having AV45-PET composite SUVR above the threshold of 1.11 (Landau et al., 2012) were determined to be amyloid positive and only amyloid positive participants were included in this study. Participants without T1 structural image in the same visit of the AV45-PET or without FDG-PET within 180 days of AV45-PET scans were excluded from the study. AV45-PET scan, structural MRI scan and neuropsychological test were conducted in the same visit. The first visit fulfilling the requirements described above was treated as the baseline visit for each individual. Seventeen participants were excluded in the imaging preprocessing steps because of failed segmentation, coregistration, or normalization. In all, 602 of the available 1,344 participants met with the inclusion criteria, including 116 CN, 314 MCI and 172 AD dementia. The acquisition date discrepancy between FDG-PET and AV45-PET scans were 2.7 ± 16.7 (mean ± standard deviation) days. Among these participants, 229 of them had follow-up FDG PET available, including 55 CN, 139 MCI, and 35 AD dementia (Figure 1 and Table 1). Baseline diagnosis was used to categorize participants in the longitudinal analysis. The diagnosis at the followup visit was not considered in the analysis.

**Table 1:** Demographic characteristics.

	CN	MCI	AD	*p* value
**(A)** Participants in the baseline analysis.				
# of subjects	116	314	172	NA
Age, y	76.1 (6.0)	73.8 (6.9)	74.5 (8.0)	0.014652
Sex, F/M	75/41	138/176	79/93	0.000506
Education, y	16.2 (2.6)	16.0 (2.8)	15.7 (2.7)	0.390729
MMSE	28.9 (1.1)	27.6 (1.9)	22.6 (3.0)	3.8E-73
MOCA	25.4 (2.5)	22.8 (3.2)	16.9 (4.6)	3.6E-54
APOE4, +/−	49/67	206/108	128/44	1.78E-08
**(B)** Participants having follow-up FDG-PET scan available for longitudinal analysis.				
# of subjects	55	139	35	NA
Age, y	77.6 (5.6)	72.8 (7.1)	74.6 (7.2)	6.17E-05
Sex, F/M	30/25	57/82	19/16	0.137522
Education, y	15.9 (2.8)	16.1 (2.8)	15.6 (3.0)	0.749848
MMSE	28.7 (1.3)	27.8 (1.8)	22.3 (2.9)	2.5E-21
MOCA	24.9 (2.5)	23.5 (3.0)	17.3 (4.6)	1.8E-15
APOE4, +/−	26/29	88/51	31/4	0.001245
Years between scans	2.2 (0.7)	2.5 (1.3)	2.1 (0.6)	0.408402

The diagnosis is based on the baseline visit. The *p* values were calculated from the chi-squared test for categorical measures or from Kruskal-Wallis test for continuous measures.

### Image acquisition and analysis

High-resolution T1 structural MRI images were acquired using MPRAGE sequence for generating gray matter mask and spatial normalization to common Montreal Neurological Institute (MNI) template. The FDG-PET scans preprocessed through coregistration, averaging, standardizing image and voxel size, and smoothing to a uniform isotropic resolution were retrieved from ADNI Laboratory of Neuroimaging (LONI) database. A detailed description of the procedure can be found at ADNI website.^2^ All FDG-PET images were first coregistered to their respective T1 structural MRI images and subsequently warped into a 2 mm MNI152 template space using Advanced Normalization Tools (ANTs)^3^. FDG-PET SUVRs were then calculated by normalizing to the average signal intensity of pons (Sperling et al., 2014). Ninety-four cortical and subcortical regions in the cerebrum from the revised automated anatomical labeling (AAL) atlas (Rolls et al., 2015) were selected as our predefined regions of interest (ROIs). The voxels within the intersection of the atlas and individual gray matter mask were averaged to calculate regional SUVR. The progression rate of regional glucose metabolism was defined as the relative annual change of SUVR between follow-up and baseline visits with an arbitrary scaling factor of 100:


(1) $$\begin{array}{l} \mathrm{FDG-PET}\;\mathrm{progression rate}=\\ 100\times \frac{{\mathrm{SUVR}}_{\mathrm{followup}}-{\mathrm{SUVR}}_{\mathrm{baseline}}\;}{\mathrm{years between scans}\times {\mathrm{SUVR}}_{\mathrm{baseline}}} \end{array}$$

A negative value of the progression rate with a larger magnitude indicates more rapid reduction of glucose metabolism.

### Cognitive assessment

The clinical dementia rating (CDR) interview was conducted for cognitive and functional assessment. The CDR sum of boxes (CDR-SB) score is derived from the CDR interview to stage dementia severity. CDR-SB, instead of MOCA or MMSE, was used to calculate cognitive decline rate, due to its larger range and variability. The rate was calculated as the annual progression of CDR-SB between follow-up and baseline visits with the equation:


(2) $$\begin{array}{l} \mathrm{Rate of cognitive decline}=\\ \left({\mathrm{CDR-SB}}_{\mathrm{followup}}-{\mathrm{CDR-SB}}_{\mathrm{baseline}}\right)/\mathrm{years between assessments}. \end{array}$$

A positive larger value of the metric indicates faster cognitive decline, and a negative value indicates improved cognition.

### Statistical analysis

Group comparisons of demographic characteristics for participants in the cross-sectional and longitudinal data are shown in Table 1. Chi-squared test was used to test the significance of group differences for categorical characteristics such as gender and apolipoprotein E (APOE) ε4 genotype (0: no ε4 allele; 1: at least 1 ε4 allele). Kruskal-Wallis test was used to conduct the group comparisons of continuous measurements, such as age, education, and Mini-Mental State Exam (MMSE) scores. With the baseline FDG-PET data, Kruskal-Wallis test was used to compare the group differences of the regional SUVR, after adjusting the influence of confounding factors, such as age, education, APOE status, and gender. Bonferroni correction was used to control for multiple comparisons in the region-wise analyses. Considering that all participants were under AD pathology (amyloid positive) but at different clinical stages, a group comparison of the FDG-PET progression rate between these groups could provide insight if hypometabolism accelerates, or slows down, or progresses constantly with disease progression. With the significant regions in the cross-sectional analysis (corrected *p* < 0.05), we then examined their progression rates with Kruskal-Wallis test, after adjusting the influence of time gap between FDG-PET scans, together with age, education, APOE status, and gender. The adjusted regional SUVRs and the progression rates were verified to be normally distributed by the Kolmogorov–Smirnov test. Then *post-hoc* 2-sample *t*-tests were carried out with the contrast MCI – CN, AD – MCI, and AD – CN at the significant ROIs identified from the Kruskal-Wallis tests. With hippocampus observed to experience hypometabolism in the cross-sectional analysis (see Result), we carried out a separate Kruskal-Wallis test on hippocampal FDG-PET SUVR after regressing out the effect of hippocampal volume, to examine if the observed hypometabolism is beyond the pathological change explained by hippocampal atrophy.

To determine if glucose metabolism is predictive of the cognitive decline among the cognitively impaired participants (MCI or AD), we assessed the association of FDG-PET data with the cognitive decline rate. The association analysis was limited to the significant regions and conducted for each region separately. Pearson’s correlation was used to evaluate the association of cognitive decline rate with regional FDG-PET SUVR (using cross-sectional data) or the progression rate (using the longitudinal data). Linear regression analysis was applied to examine the association with both the baseline FDG-PET SUVR and its progression rate as independent variables (Cognitive decline rate ~ 1 + FDG-PET SUVR + FDG progression rate). The adjusted R squared value from linear regression analysis was used to assess the association between FDG-PET data and cognitive decline rate.

## Results

### Group differences of regional glucose metabolism between diagnostic groups

**Figure 2:**
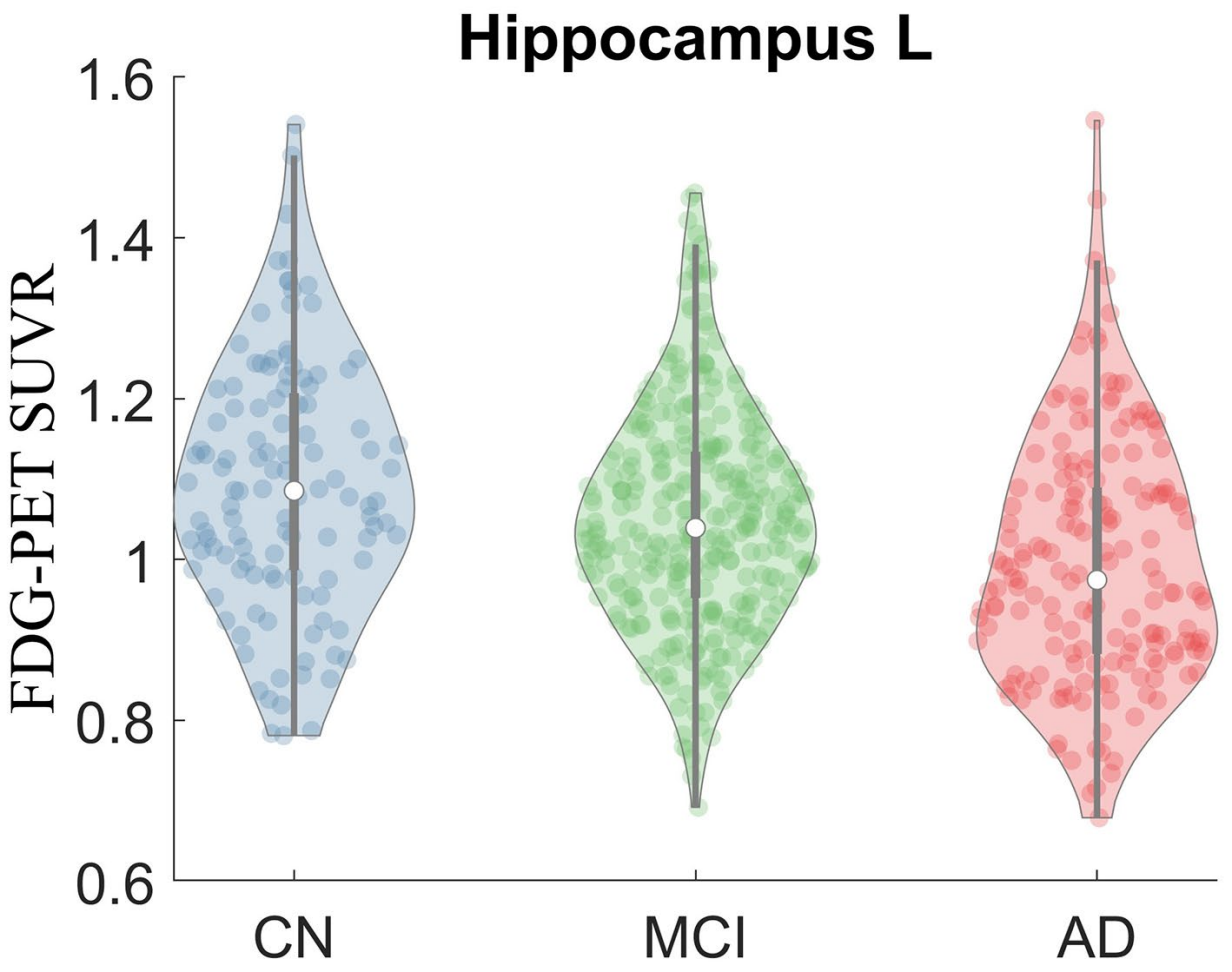
Violin plot of FDG-PET SUVR in CN, MCI and AD at left hippocampus. Significant group difference was identified in Kruskal-Wallis test (*p* = 2.17E-7).

Kruskal-Wallis test was carried out on baseline FDG-PET data to examine group differences; significant differences were found between diagnostic groups after Bonferroni correction. The full list of regions can be found in Supplementary Table 1. For simplicity, only the significant regions with at least medium effect (|cohen’s d| ≥ 0.5) in the group comparison between CN and AD are shown in Table 2 and presented in the following. The original *p* values from Kruskal-Wallis test (5th column) are listed in the table. Significant regional differences between groups were found at bilateral posterior cingulate cortex, bilateral precuneus and regions in parietal lobe and temporal lobe, including left hippocampus (Figure 2), bilateral parahippocampal gyrus, bilateral inferior parietal lobule, bilateral angular gyrus, bilateral superior temporal lobe, bilateral middle temporal lobe and bilateral inferior temporal lobe, left superior temporal pole and left middle temporal pole. All these regions had the lowest SUVRs in the AD dementia group, intermediate values in MCI group, and the highest values in the CN group. No region showed higher FDG in the cognitively impaired (either MCI or AD) groups than the CN individuals. The observed hypometabolism at left hippocampus remained to be significant after regressing out hippocampal volume (*p* = 0.0062 in Kruskal-Wallis test, see Supplementary Figure 1).

**Table 2:** Brain regions identified to have significantly different glucose metabolism between CN, MCI and AD in the baseline analysis.

Region	CN	MCI	AD	*p* value
Cingulate Post L	1.63 (1.60, 1.66)	1.59 (1.57, 1.61)	1.45 (1.42, 1.48)	4.79E-18
Cingulate Post R	1.64 (1.61, 1.66)	1.60 (1.58, 1.61)	1.48 (1.45, 1.51)	4.41E-14
Hippocampus L	1.09 (1.06, 1.12)	1.05 (1.03, 1.06)	1.00 (0.97, 1.02)	2.17E-07
ParaHippocampal L	1.12 (1.10, 1.14)	1.08 (1.07, 1.09)	1.01 (1.00, 1.03)	5.86E-15
ParaHippocampal R	1.13 (1.11, 1.16)	1.10 (1.09, 1.12)	1.05 (1.03, 1.07)	7.45E-08
Occipital Inf L	1.39 (1.35, 1.43)	1.40 (1.37, 1.42)	1.28 (1.25, 1.32)	1.21E-07
Parietal Inf L	1.36 (1.33, 1.39)	1.36 (1.34, 1.37)	1.26 (1.23, 1.29)	4.03E-09
Parietal Inf R	1.40 (1.36, 1.43)	1.38 (1.37, 1.40)	1.27 (1.24, 1.30)	5.42E-10
Angular L	1.34 (1.31, 1.37)	1.32 (1.30, 1.34)	1.19 (1.16, 1.23)	6.64E-12
Angular R	1.35 (1.32, 1.38)	1.34 (1.32, 1.36)	1.23 (1.20, 1.26)	1.43E-10
Precuneus L	1.54 (1.51, 1.57)	1.53 (1.51, 1.55)	1.44 (1.41, 1.46)	2.4E-08
Precuneus R	1.55 (1.52, 1.58)	1.54 (1.52, 1.55)	1.46 (1.43, 1.48)	3.78E-07
Temporal Sup L	1.29 (1.27, 1.31)	1.27 (1.26, 1.29)	1.22 (1.19, 1.24)	3.2E-06
Temporal Sup R	1.32 (1.30, 1.35)	1.31 (1.29, 1.32)	1.25 (1.22, 1.27)	2.41E-06
Temporal Pole Sup L	1.13 (1.11, 1.15)	1.10 (1.09, 1.12)	1.05 (1.02, 1.07)	4.36E-08
Temporal Mid L	1.34 (1.31, 1.36)	1.30 (1.28, 1.32)	1.17 (1.14, 1.20)	1.44E-19
Temporal Mid R	1.34 (1.32, 1.37)	1.32 (1.30, 1.33)	1.22 (1.19, 1.24)	6.18E-13
Temporal Pole Mid L	1.07 (1.04, 1.10)	1.04 (1.02, 1.06)	0.98 (0.96, 1.01)	1.48E-06
Temporal Inf L	1.31 (1.28, 1.34)	1.30 (1.28, 1.32)	1.17 (1.14, 1.20)	4.13E-16
Temporal Inf R	1.36 (1.34, 1.39)	1.34 (1.32, 1.35)	1.24 (1.22, 1.27)	1.17E-11

All the regions have passed the Bonferroni correction over multiple comparison in the Kruskal-Wallis tests. The mean value were shown for each group with the 95% confidence intervals of the mean values listed in the bracket. The original p values from Kruskal-Wallis tests (5th column) were reported in the table. For simplicity, only the significant ROIs with the additional requirement of at least medium effect in the contrast AD –CN (|cohen’s| > =0.5) were listed in the table. A complete list of significant ROIs and the *post-hoc* statistical analysis can be seen in Supplementary Table 1.

**Figure 3:**
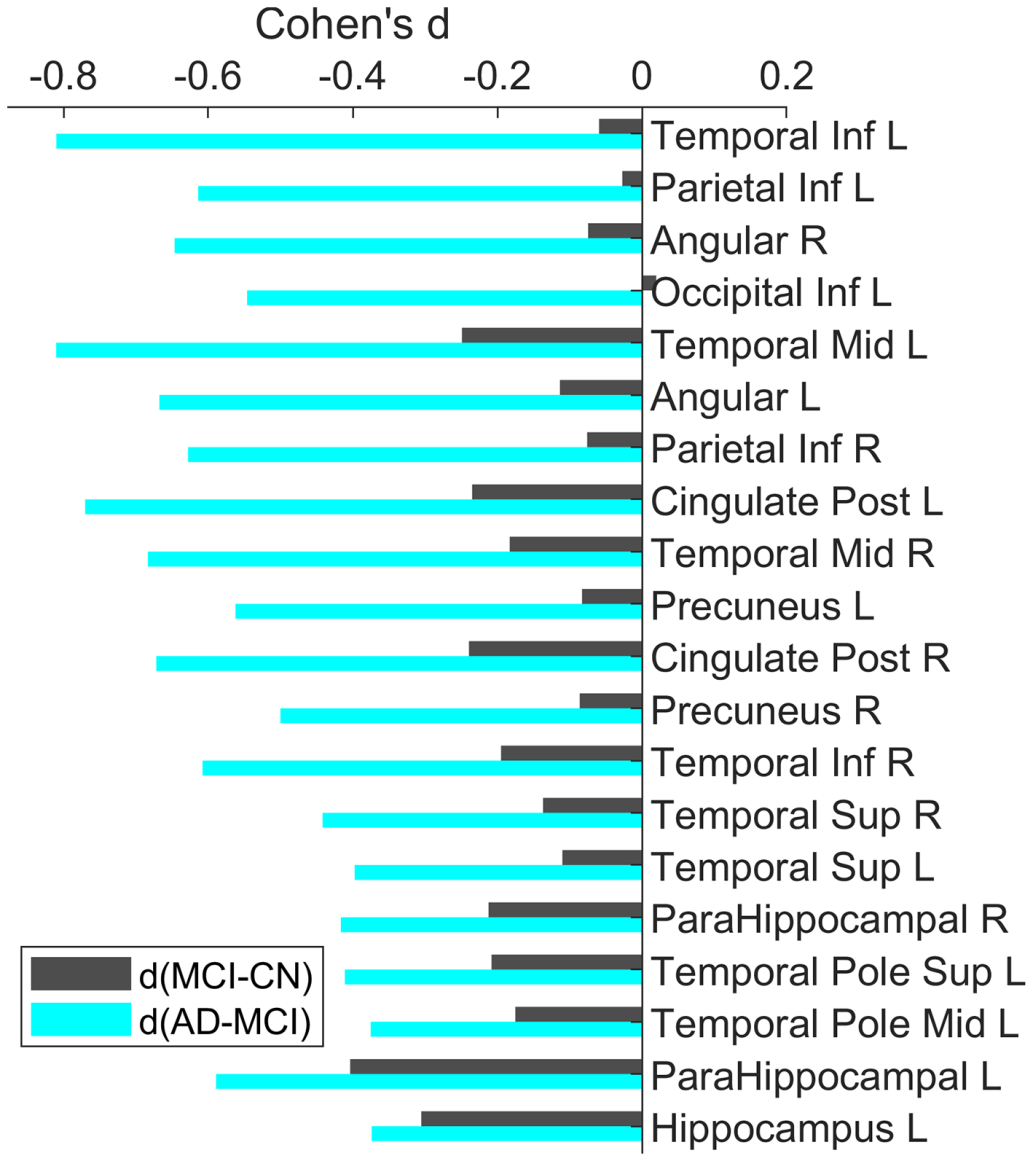
Bar plots of the effect sizes for the contrast MCI-CN and AD-MCI using baseline FDG-PET data. All these ROIs were identified to have significant group differences. The ROIs were sorted based on the difference of the effect sizes for the two contrasts. Across all these ROIs, the group differences between AD and MCI had larger effect than the differences between MCI and CN.

The bar plots of the effect sizes from the *post-hoc* two-sample *t*-test for the contrasts MCI – CN (black) and AD – MCI (green) are shown in Figure 3, sorted by the discrepancy of the effect size of the two contrasts. Although MCI group overall had lower regional SUVR than CN group across all ROIs, the differences between MCI and CN had only small or very small effect sizes (|Cohen’s d| < 0.5), with the most substantial effect observed in the left parahippocampal gyrus (Cohen’s *d* = −0.40). In contrast, most of the significantly different regions between AD dementia and MCI had group differences with at least medium effect sizes (|Cohen’s d| ≥ 0.5), the most substantial difference was observed in the left middle temporal lobe (Cohen’s *d* = −0.81). Overall, the differences of glucose metabolism between AD dementia and MCI were larger than the differences between MCI and CN, while the differences of AV45-PET between AD dementia and MCI were weaker than the differences between MCI and CN (Supplementary Table 2). After adjusting for AV45-PET composite SUVR and the time duration since cognitive symptom onset as covariates, more substantial metabolic differences between MCI and AD dementia were still observed, compared to the differences between CN and MCI (Supplementary Table 3). We also assessed the role of the APOE on regional FDG-PET SUVR at these significantly different regions and found the influence of APOE was marginal (Supplementary Figure 2).

### Faster reduction of glucose metabolism at the later clinical stage

With the observation in the baseline that the group differences between MCI and AD were more substantial than the differences between CN and MCI even after adjusting the time duration since the cognitive symptom onset, we tested the FDG-PET progression rates between groups at the significant regions from cross-sectional analysis to evaluate how glucose hypometabolism progresses with disease progression. Bilateral posterior cingulate cortex, left inferior parietal lobe, bilateral angular gyrus, and right middle temporal lobe showed significantly different progression rates between groups after Bonferroni correction. Thirteen regions were identified to show the differences of progression rates between AD and CN with at least medium effect, including bilateral posterior cingulate cortex, bilateral inferior parietal lobe, bilateral angular gyrus, bilateral precuneus, bilateral superior temporal lobe, bilateral middle temporal lobe and right inferior temporal lobe (Table 3). Among these regions, fastest decline was observed in the AD dementia group. Reduction of regional glucose metabolism was also observed in MCI group but with slower rate. The CN group did not show the trend of hypometabolism at the group level. Similar findings were observed when the absolute instead of relative progression rate was used with the definition of absolute progression rate = (SUVR_followup_−SUVR_baseline_)/years between scans (see Supplementary Table 4).

**Table 3:** Comparisons of FDG-PET progression rates between CN, MCI and AD in the longitudinal analysis.

Region	CN	MCI	AD	*p* value		Cohen’s d	
					MCI – CN	AD – MCI	AD – CN
Cingulate Post L	0.5 (−0.9, 1.9)	−0.5 (−1.2, 0.1)	−3.3 (−5.0, −1.6)	0.000321	−0.24	−0.68	−0.75
Cingulate Post R	0.7 (−0.7, 2.1)	−0.4 (−1.0, 0.3)	−3.0 (−4.7, −1.2)	0.000596	−0.26	−0.63	−0.72
Parietal Inf L	0.4 (−1.0, 1.8)	−0.4 (−1.1, 0.3)	−4.2 (−6.4, −2.0)	0.000398	−0.19	−0.81	−0.80
Parietal Inf R	0.7 (−0.8, 2.2)	−0.8 (−1.6, 0.1)	−3.8 (−6.0, −1.6)	0.004091	−0.29	−0.59	−0.76
Angular L	0.4 (−1.3, 2.0)	−1.0 (−1.8, −0.1)	−4.9 (−6.9, −2.8)	6.63E-05	−0.26	−0.72	−0.86
Angular R	0.6 (−1.0, 2.2)	−0.5 (−1.4, 0.4)	−3.7 (−5.7, −1.7)	0.002245	−0.20	−0.58	−0.73
Precuneus L	0.6 (−0.8, 2.0)	−0.4 (−1.0, 0.2)	−2.9 (−4.7, −1.1)	0.004631	−0.24	−0.61	−0.68
Precuneus R	0.5 (−0.9, 1.9)	−0.4 (−1.1, 0.2)	−2.4 (−4.1, −0.7)	0.023856	−0.22	−0.49	−0.57
Temporal Sup L	0.6 (−0.6, 1.8)	−0.8 (−1.4, −0.1)	−1.9 (−3.7, −0.1)	0.065792	−0.32	−0.27	−0.51
Temporal Sup R	0.7 (−0.5, 1.9)	−0.9 (−1.5, −0.3)	−2.2 (−3.8, −0.6)	0.028404	−0.41	−0.32	−0.63
Temporal Mid L	0.3 (−1.0, 1.6)	−1.3 (−2.0, −0.6)	−3.1 (−4.9, −1.4)	0.007434	−0.38	−0.44	−0.69
Temporal Mid R	0.8 (−0.5, 2.2)	−1.1 (−1.8, −0.5)	−3.5 (−5.1, −1.8)	0.000683	−0.47	−0.55	−0.88
Temporal Inf R	−0.4 (−2.0, 1.2)	−1.0 (−1.7, −0.2)	−3.5 (−5.0, −2.0)	0.003161	−0.12	−0.57	−0.59

The mean value were shown for each group with the 95% confidence intervals of the mean values listed in the bracket. The original p values from Kruskal-Wallis tests and the effect sizes from *post-hoc* statistics were reported in the table. Only the regions with at least medium effect in the contrast AD – CN were included.

### Lower regional glucose metabolism at baseline (and faster metabolic decline) was associated with more rapid cognitive decline

**Figure 4:**
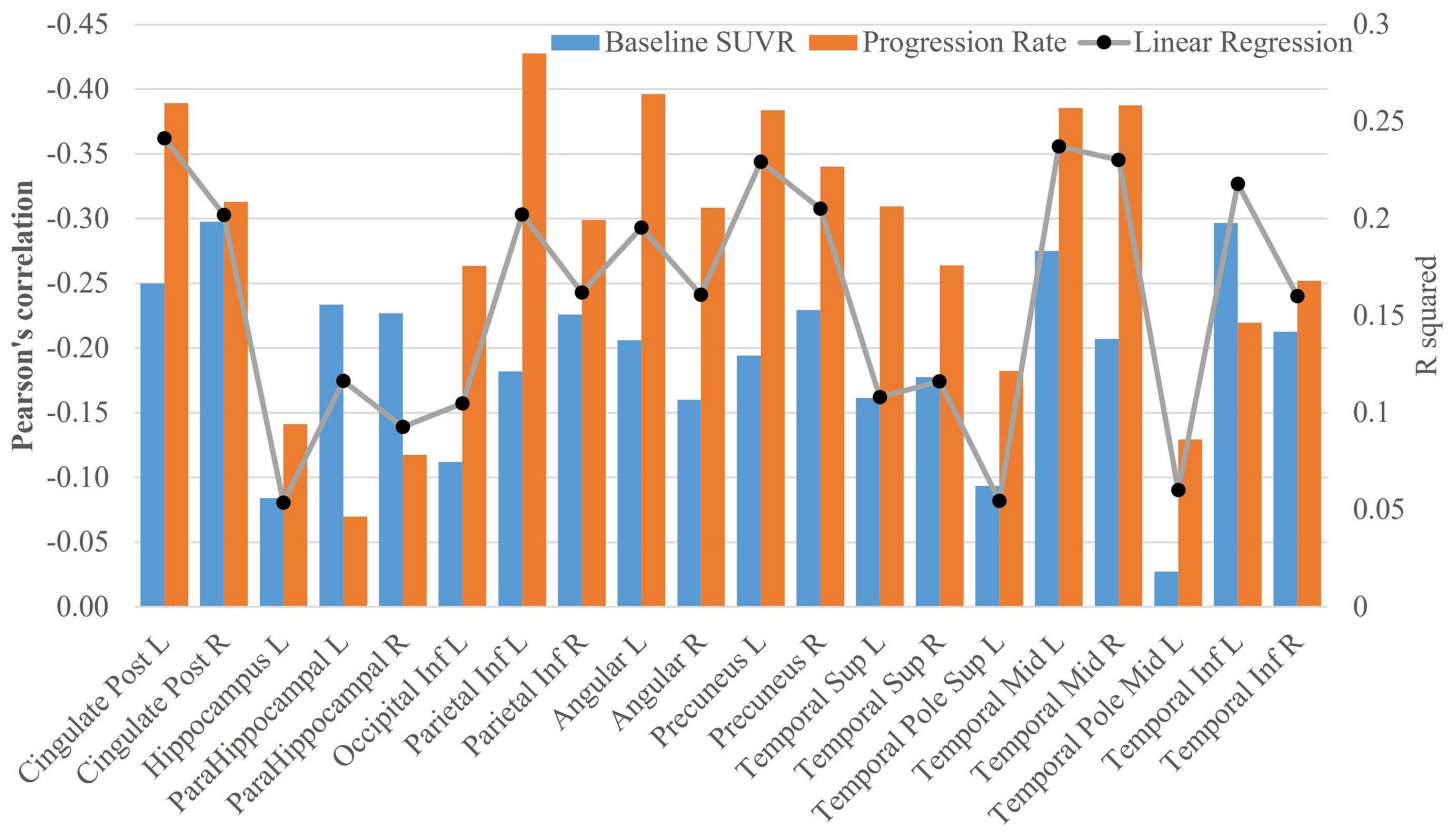
Association analysis of FDG-PET data with cognitive decline rate. The bar plots of Pearson’s correlations between cognitive decline rate and SUVR at baseline or FDG-PET progression rate were shown in the figure. The adjusted *R*^2^ values (black dots) from the linear regression model Cognitive decline rate ~ 1 + baseline SUVR + FDG-PET progression rate were also plotted in the figure.

Pearson’s correlation was used to characterize the association of cognitive decline rate with baseline FDG-PET SUVR or its progression rate (see Figure 4). Except left superior temporal pole, left middle temporal pole and left hippocampus, all the other regions had FDG-PET SUVR and (or) the progression rate significantly correlated with the rate of cognitive decline (only significant ROIs shown in Figure 4; the significance levels marked in Supplementary Table 5). The correlations with the progression rate overall had larger magnitude than the correlation with the FDG-PET SUVR, with the exceptions at bilateral parahippocampus and left inferior temporal lobe. The strongest association with regional SUVR was observed at the right posterior cingulate cortex (*r* = −0.30, *p* = 2.58×10–6), and the strongest association with progression rate was observed at the left inferior parietal lobe (*r* = −0.43, *p* = 4.03×10–9). In the linear regression model Cognitive decline rate ~ 1 + baseline SUVR + FDG-PET progression rate, left posterior cingulate cortex had the most significant fitting (FDG-PET SUVR: *t* = −4.7, *p* = 4.5×10–6, FDG-PET progression rate: *t* = −6.1, *p* = 7.06×10–9; *F*_2,171_ = 28.5, adjusted *R*^2^ = 0.24 and *p* = 2.06E-11, see Figure 4). The adjusted *R*^2^ values (black dots) from the linear regression model can be found in Figure 4.

## Discussion

In this study, cross-sectional and longitudinal analysis were carried out with FDG-PET data to evaluate brain glucose metabolism and its progression rate with disease progression among a large cohort on the Alzheimer’s continuum as shown by a positive amyloid PET scan. In addition, we assessed the potential prognostic value of longitudinal FDG-PET scans in predicting cognitive decline.

The cross-sectional analysis showed that the AD group had abnormally low glucose metabolism in extensive brain regions, these regions largely overlapped with the commonly reported parieto-temporal area among participants with probable AD in early FDG-PET studies, who were clinically diagnosed as dementia without confirmed amyloid status (De Santi et al., 2001; Herholz et al., 2002; Mosconi et al., 2008a). Despite the crucial role of hippocampus and parahippocampal gyrus in AD, these regions were not detected to experience hypometabolism in probable AD, even with a large cohort (Herholz et al., 2002). A progression of deficits in hippocampal metabolism were observed with longitudinal FDG-PET scans from two cognitively normal elderly who had post-mortem diagnosis of definite AD and progressed to dementia in the followup visits (Mosconi et al., 2009), but whether this finding can be generalized from the very few participants to the general AD population needs to be verified with a larger sample size.

With the assistance of AV45-PET scan to exclude participants not under AD pathology, we found that the AD dementia group had significantly lower hippocampal metabolism than the prodromal and preclinical stage of AD dementia (the MCI and CN groups in the study), suggesting that hypometabolism at hippocampus may be traditionally underestimated due to participant heterogeneity induced by ambiguous amyloid status. An alternative factor which could lead to discrepant findings is the different clinical stages of the participants enrolled in these studies. The middle temporal lobe was observed to experience hypermetabolism instead of hypometabolism at the early stage of the disease (Apostolova et al., 2018), which might contribute to the metabolic differences between AD dementia and MCI or CN in our study. The metabolic difference between CN, MCI and AD at hippocampus was weaker but remained to be significant even after adjusting hippocampal volume. Another ADNI study demonstrated that the hypometabolism is a biomarker independent from hippocampal atrophy in characterizing neurodegeneration in AD (Ou et al., 2019). The complementary role of hippocampal volume and glucose metabolism for neurodegeneration is in line with the relevant but differentiated biological mechanisms behind hypometabolism (reduced glucose metabolism) and atrophy (neuronal loss).

Abnormal β-amyloid accumulation is hypothesized to be the initial pathological change experienced decades before an individual is diagnosed as having symptomatic AD (Sperling et al., 2014), and it plateaus in the later stage of the disease (Villemagne et al., 2013). The amyloid progression model is consistent with our observation that β-amyloid difference was substantial between MCI and CN but negligible between AD and MCI for most regions. In this study, longitudinal data were used to investigate the progression rate of regional glucose metabolism among participants along the AD continuum, allowing us to confirm whether glucose metabolism deficit, similar to β-amyloid, slows down at the later disease stage, or accelerates with disease progression. The AD dementia group was found to have glucose metabolism decline most rapidly compared to the CN and MCI groups. Glucose metabolism deficit was also observed at the prodromal stage of AD (MCI) with a slower rate but the trend of hypometabolism was not observed before the onset of cognitive symptoms (namely the CN group). Consistent with the longitudinal analyses, the group differences between AD dementia and MCI evident in the baseline data were more substantial compared to the group differences between MCI and CN. These observations suggest that hypometabolism progresses more rapidly in the later stages of the disease. The participants with normal cognition did not show a trend toward hypometabolism in these regions, even though they had abnormal brain amyloid, consistent with the hypothesis that neurodegeneration follows amyloid deposition after a time lag (Jack et al., 2010). The time lag and distinct progression curves of glucose metabolism and brain amyloid in the AD continuum may explain discrepant observations on the relationship of FDG to amyloid PET (Edison et al., 2007; Furst et al., 2012; Lowe et al., 2014; Altmann et al., 2015), the association may depend on the disease stage of the participants under investigation.

The longitudinal analysis demonstrated the capability of FDG-PET to image the progression of brain metabolism with a time gap of approximately 2 years, suggesting the potential value of collecting longitudinal FDG-PET data for assessing the progression of neurodegeneration. Both FDG-PET SUVR at baseline and its progression rate were significantly associated with the cognitive decline rate for most of the significant regions identified with baseline data. Faster cognitive decline was shown to be correlated to lower glucose metabolism at parietal lobe and temporal lobe with mild-to-moderate correlations, which is consistent with the prognostic value of a single FDG-PET scan in predicting clinical stability and disease progression reported in previous studies (Mosconi et al., 2004; Anchisi et al., 2005; Iaccarino et al., 2019). More importantly, compared to the magnitude of FDG-PET, we demonstrated that the progression rate of longitudinal FDG-PET signal was more strongly correlated to cognitive decline with moderate-to-large correlations. Moreover, we also noticed the regional heterogeneity in the association analysis. Different from other regions, the FDG-PET SUVRs and the progression rates at left hippocampus and left middle temporal role were barely associated with cognitive decline rate. The CDR-SB used in the analysis represents a general assessment of cognition, the insignificant associations at these regions might be because their glucose metabolic deficits are specifically related to a certain cognitive domain. More investigations would be required to clarify the domain-specific glucose hypometabolism. Collectively, these findings suggest that FDG-PET, particularly the collection of longitudinal scans, may serve as a pharmacokinetic biomarker to assess if a disease-modifying agent could slow cognitive decline.

There are several limitations in our study. First, following the amyloid hypothesis that considered amyloid deposition as the initial causal event in AD (Hardy and Higgins, 1992), all participants were required to have positive amyloid PET scan to exclude individuals without AD pathology. The amyloid hypothesis is widely adopted in current clinical trials (Jack et al., 2018) but has also been subject to criticism (Garrett, 2018; Glymour et al., 2018; Morris et al., 2018). Amyloid positive individuals with negative tau PET were demonstrated to less likely develop dementia in 5 years than those with positive tau imaging (Josephs et al., 2022), suggesting that tau pathology, in addition to glucose metabolism, could contribute to cognitive decline. However, tau pathology was not considered in our analysis due to very few subjects having tau-PET available in the same visit. Second, we demonstrated that FDG-PET was sensitive enough to detect the difference between visits with a two-year period. Such a time gap may be too long for interventional trials and further refinement of the temporal sensitivity of FDG-PET is warranted. Third, the association analysis of FDG-PET with cognitive decline was conducted at the group level, the prognostic value of FDG-PET in predicting cognitive decline at individual level requires examination.

In summary, this study investigated glucose metabolism among participants with the defining signature of AD (abnormal brain amyloid), covering patients from an early stage of the disease with normal cognition, prodromal AD (MCI), to AD dementia. The results indicate that hippocampal hypometabolism is underestimated in AD probably due to ambiguous amyloid status in early studies, the reduction of brain glucose metabolism (measured as FDG-PET SUVR) accelerates with disease progression toward later stages, and glucose metabolism and its progression rate are informative for predicting disease progression.

## Data availability statement

Publicly available datasets were analyzed in this study. This data can be found here: http://adni.loni.usc.edu/.

## Ethics statement

The studies involving human participants were reviewed and approved by the Alzheimer’s Disease Neuroimaging Initiative. The patients/participants provided their written informed consent to participate in this study.

## Author contributions

ZY, JC, JK, and DC: conceptualization and writing—review and editing. ZY, JC, and DC: methodology. ZY: formal analysis, writing—original draft, and visualization. DC: supervision. All authors read and approved the final version of the manuscript.

## Funding

This research project was supported by the NIH Grant No. 1RF1AG071566, Cleveland Clinic Keep Memory Alive Young Investigator Award, a private grant from Stacie and Chuck Matthewson, a private grant from Peter and Angela Dal Pezzo, and a private grant from Lynn and William Weidner. The data collection and sharing for this study was funded by the Alzheimer’s Disease Neuroimaging Initiative (ADNI) (National Institutes of Health Grant U01 AG024904) and DOD ADNI (Department of Defense award number W81XWH-12-2-0012). ADNI is funded by the National Institute on Aging, the National Institute of Biomedical Imaging and Bioengineering, and through generous contributions from the following: AbbVie, Alzheimer’s Association; Alzheimer’s Drug Discovery Foundation; Araclon Biotech; BioClinica, Inc.; Biogen; Bristol-Myers Squibb Company; CereSpir, Inc.; Cogstate; Eisai Inc.; Elan Pharmaceuticals, Inc.; Eli Lilly and Company; EuroImmun; F. Hoffmann-La Roche Ltd. and its affiliated company Genentech, Inc.; Fujirebio; GE Healthcare; IXICO Ltd.; Janssen Alzheimer Immunotherapy Research & Development, LLC.; Johnson &Johnson Pharmaceutical Research & Development LLC.; Lumosity; Lundbeck; Merck & Co., Inc.; Meso Scale Diagnostics, LLC.; NeuroRx Research; Neurotrack Technologies; Novartis Pharmaceuticals Corporation; Pfizer Inc.; Piramal Imaging; Servier; Takeda Pharmaceutical Company; and Transition Therapeutics. The Canadian Institutes of Health Research is providing funds to support ADNI clinical sites in Canada. Private sector contributions are facilitated by the Foundation for the National Institutes of Health (www.fnih.org). The grantee organization is the Northern California Institute for Research and Education, and the study is coordinated by the Alzheimer’s Therapeutic Research Institute at the University of Southern California. ADNI data are disseminated by the Laboratory for Neuro Imaging at the University of Southern California. JC was supported by NIGMS grant P20GM109025; NINDS grant U01NS093334; NIA grant R01AG053798; NIA grant P20AG068053; NIA grant P30AG072959; NIA grant R35AG71476; Alzheimer’s Disease Drug Discovery Foundation (ADDF); Ted and Maria Quirk Endowment; and the Joy Chambers-Grundy Endowment.

## Conflict of interest

JC has provided consultation to Acadia, Actinogen, Alkahest, AlphaCognition, AriBio, Biogen, Cassava, Cerecin, Cortexyme, Diadem, EIP Pharma, Eisai, GemVax, Genentech, Green Valley, GAP Innovations, Grifols, Janssen, Karuna, Lilly, Lundbeck, LSP, Merck, NervGen, Novo Nordisk, Oligomerix, Optoceutics, Ono, Otsuka, PRODEO, Prothena, ReMYND, Resverlogix, Roche, Sage Therapeutics, Signant Health, Suven, TrueBinding, and Vaxxinity pharmaceutical, assessment, and investment companies.

The remaining authors declare that the research was conducted in the absence of any commercial or financial relationships that could be construed as a potential conflict of interest.

## Publisher’s note

All claims expressed in this article are solely those of the authors and do not necessarily represent those of their affiliated organizations, or those of the publisher, the editors and the reviewers. Any product that may be evaluated in this article, or claim that may be made by its manufacturer, is not guaranteed or endorsed by the publisher.

## Footnotes

^1^ https://adni.loni.usc.edu/

^2^ https://adni.loni.usc.edu/methods/pet-analysis-method/pet-analysis/

^3^ https://www.nitrc.org/projects/ants

## Abbreviations

Aβ: β-amyloid
AD: Alzheimer’s disease
ADNI: Alzheimer’s Disease Neuroimaging Initiative
CDR-SB: clinical dementia rating scale sum of boxes
CN: Cognitively normal
MCI: mild cognitive impairment
ROI: region of interest
SUVR: standardized uptake value ratio
FDG: fluorodeoxyglucose
PET: positron emission tomography
ROI: region of interest
