# Supplementary figures and images for: Accelerated hypometabolism with disease progression associated with faster cognitive decline among amyloid positive patients
Source: Front Neurosci. 2023 Apr 14;17:1151820. doi: 10.3389/fnins.2023.1151820 (PMC10140339; doi:10.3389/fnins.2023.1151820)

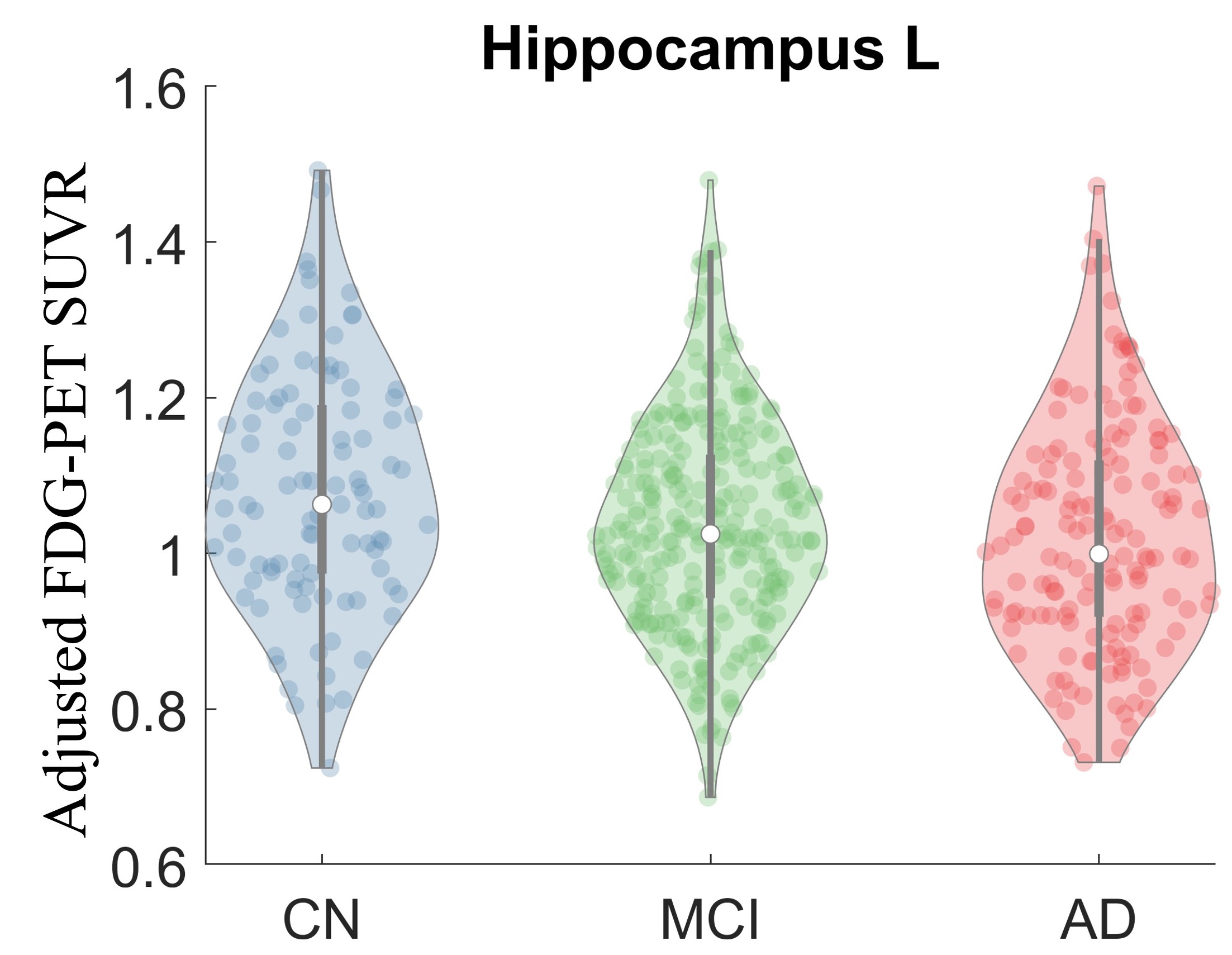

Supplement: Supplementary file 1 [file Data_Sheet_1.ZIP › Supplementary Material/Supp Figure 1 Hippo_Hippocampus L.jpg]

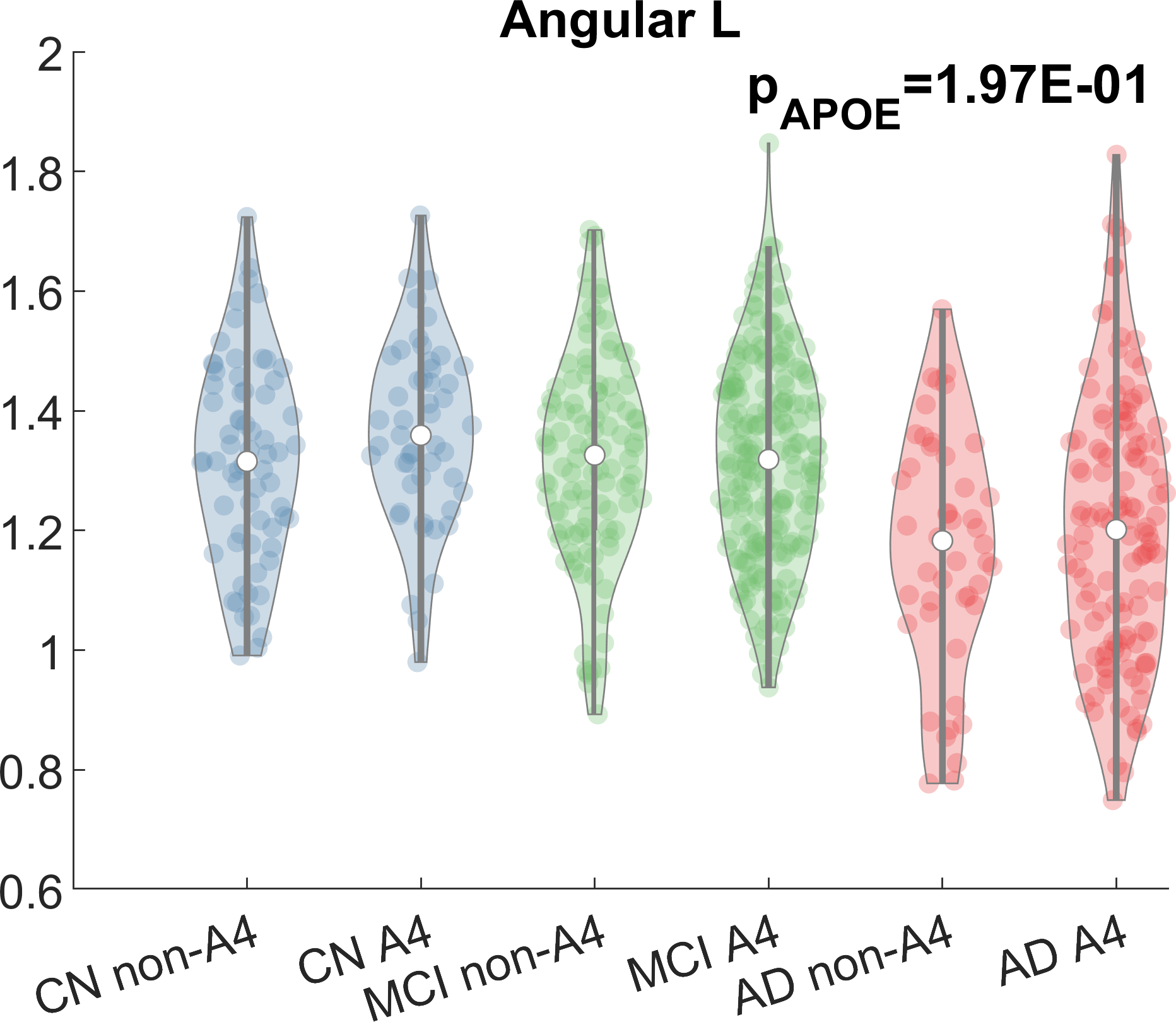

Supplement: Supplementary file 1 [file Data_Sheet_1.ZIP › Supplementary Material/Supp Figure 2/APOE4_Angular L.tif]

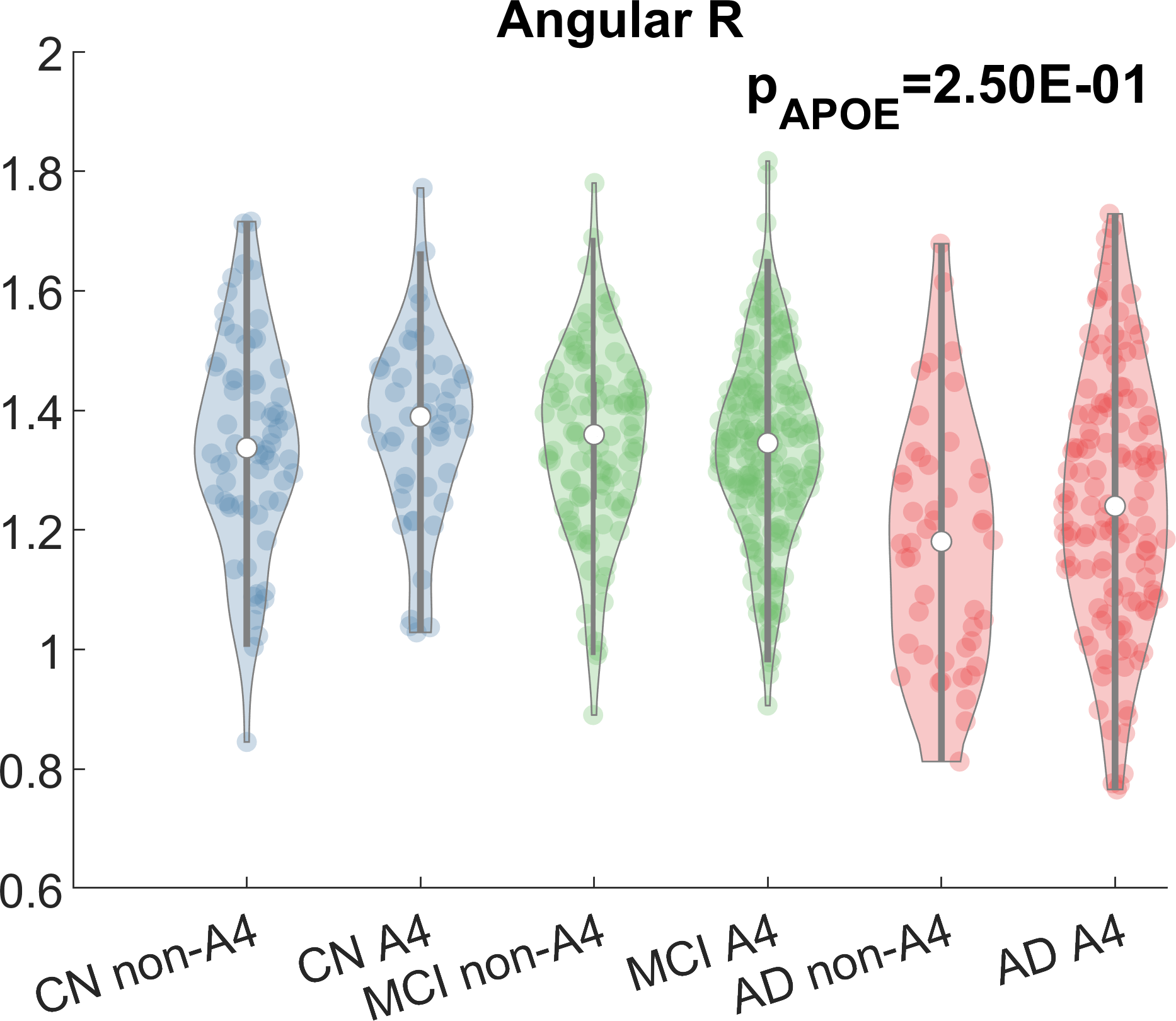

Supplement: Supplementary file 1 [file Data_Sheet_1.ZIP › Supplementary Material/Supp Figure 2/APOE4_Angular R.tif]

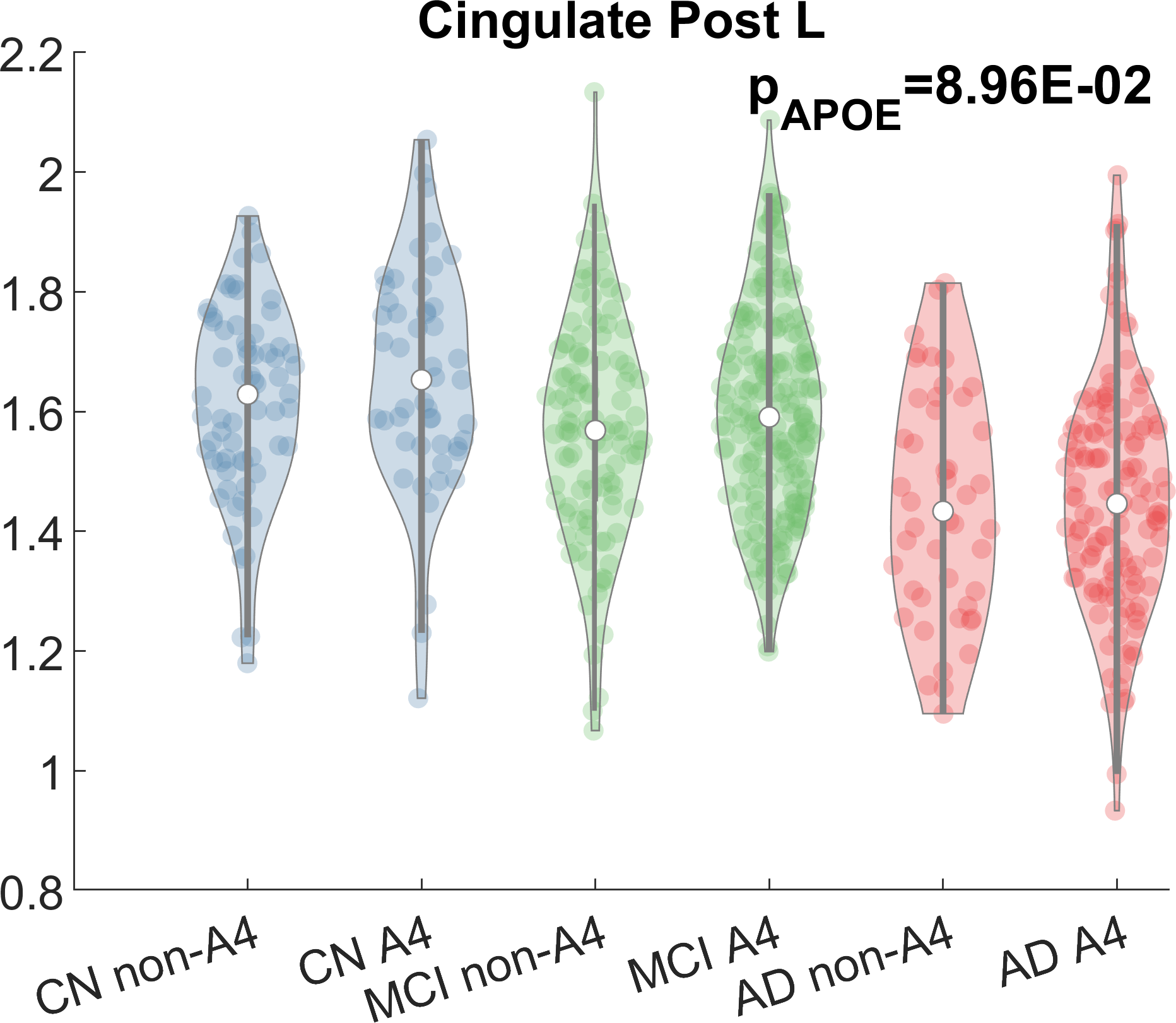

Supplement: Supplementary file 1 [file Data_Sheet_1.ZIP › Supplementary Material/Supp Figure 2/APOE4_Cingulate Post L.tif]

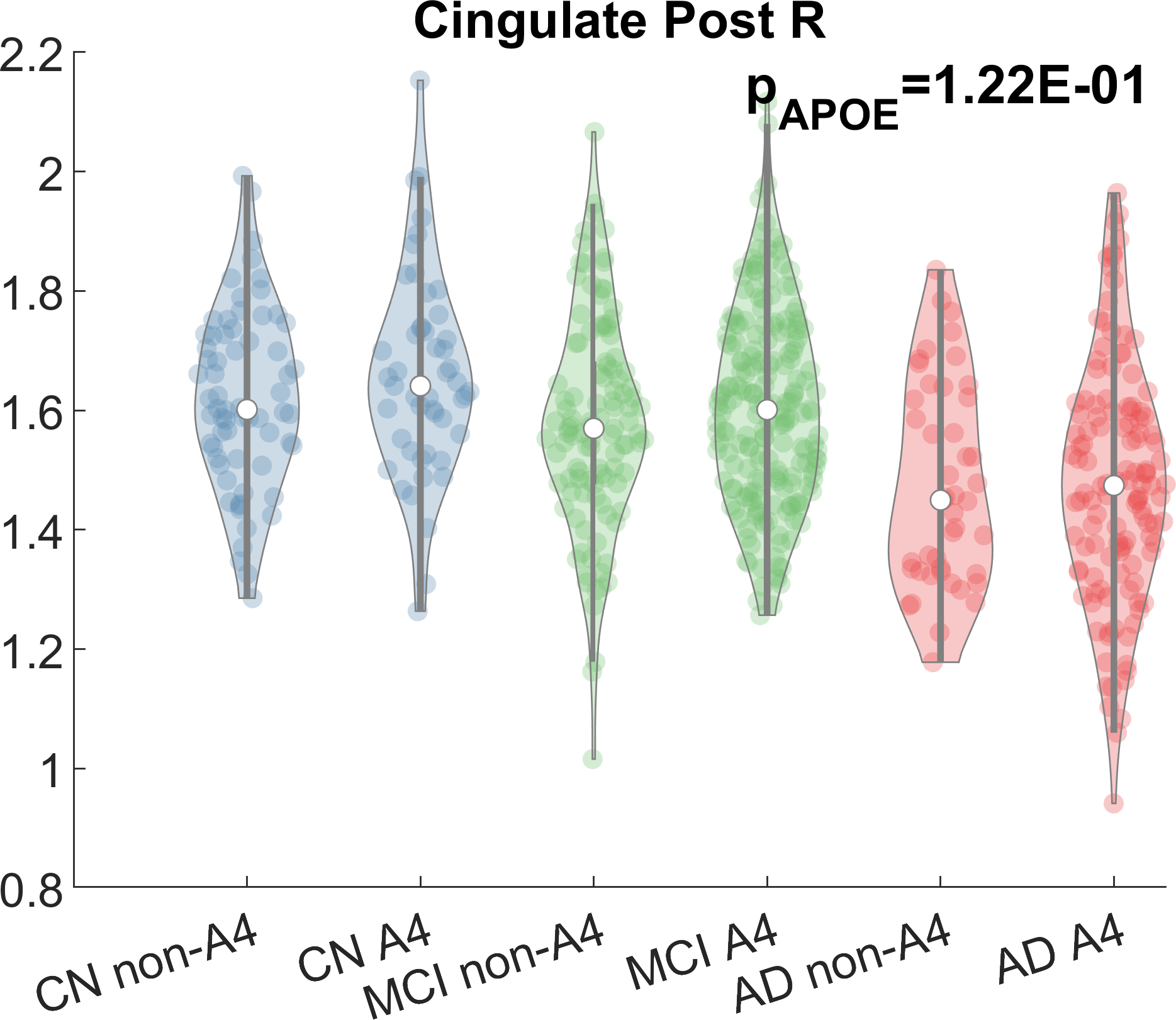

Supplement: Supplementary file 1 [file Data_Sheet_1.ZIP › Supplementary Material/Supp Figure 2/APOE4_Cingulate Post R.tif]

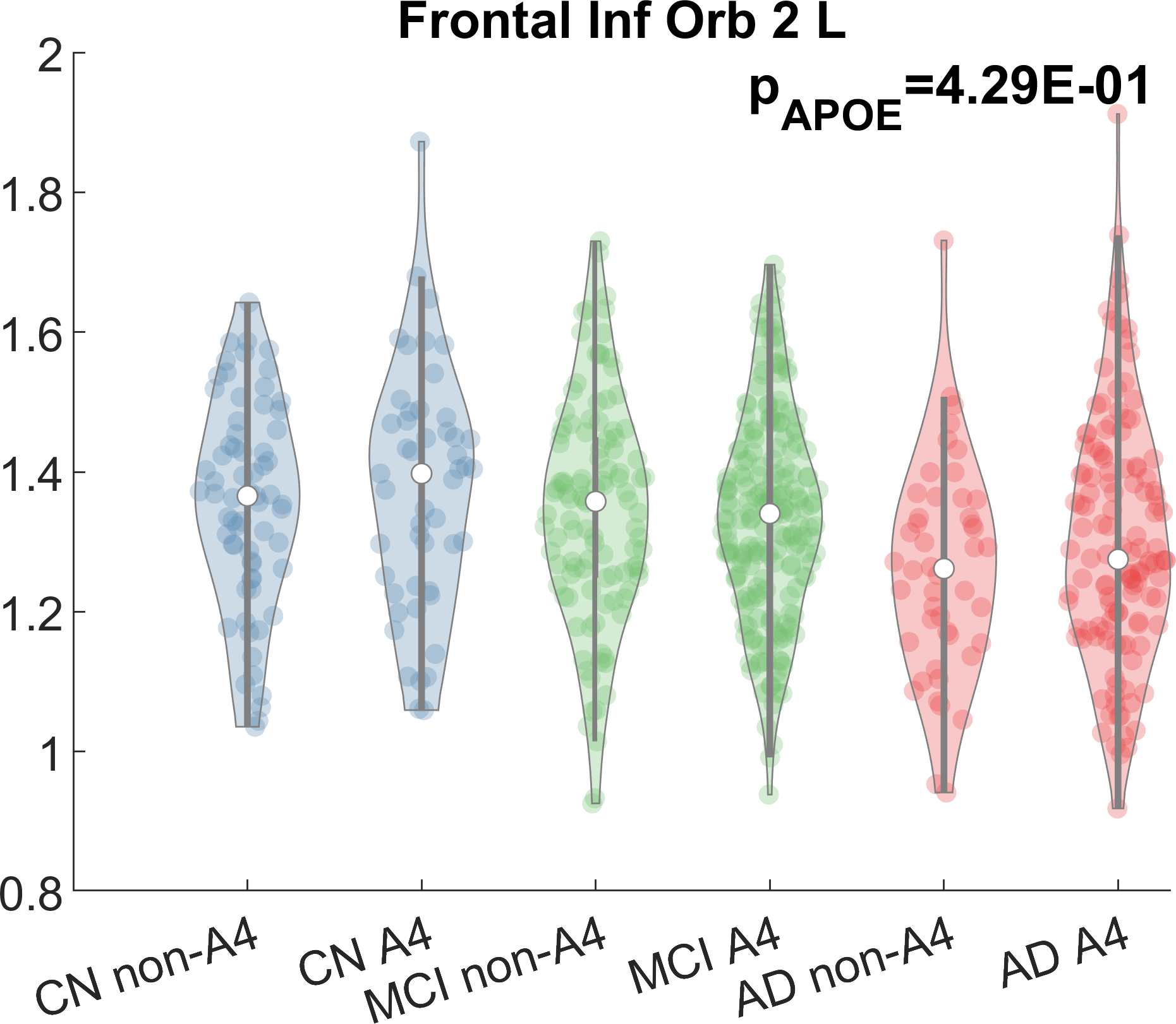

Supplement: Supplementary file 1 [file Data_Sheet_1.ZIP › Supplementary Material/Supp Figure 2/APOE4_Frontal Inf Orb 2 L.tif]

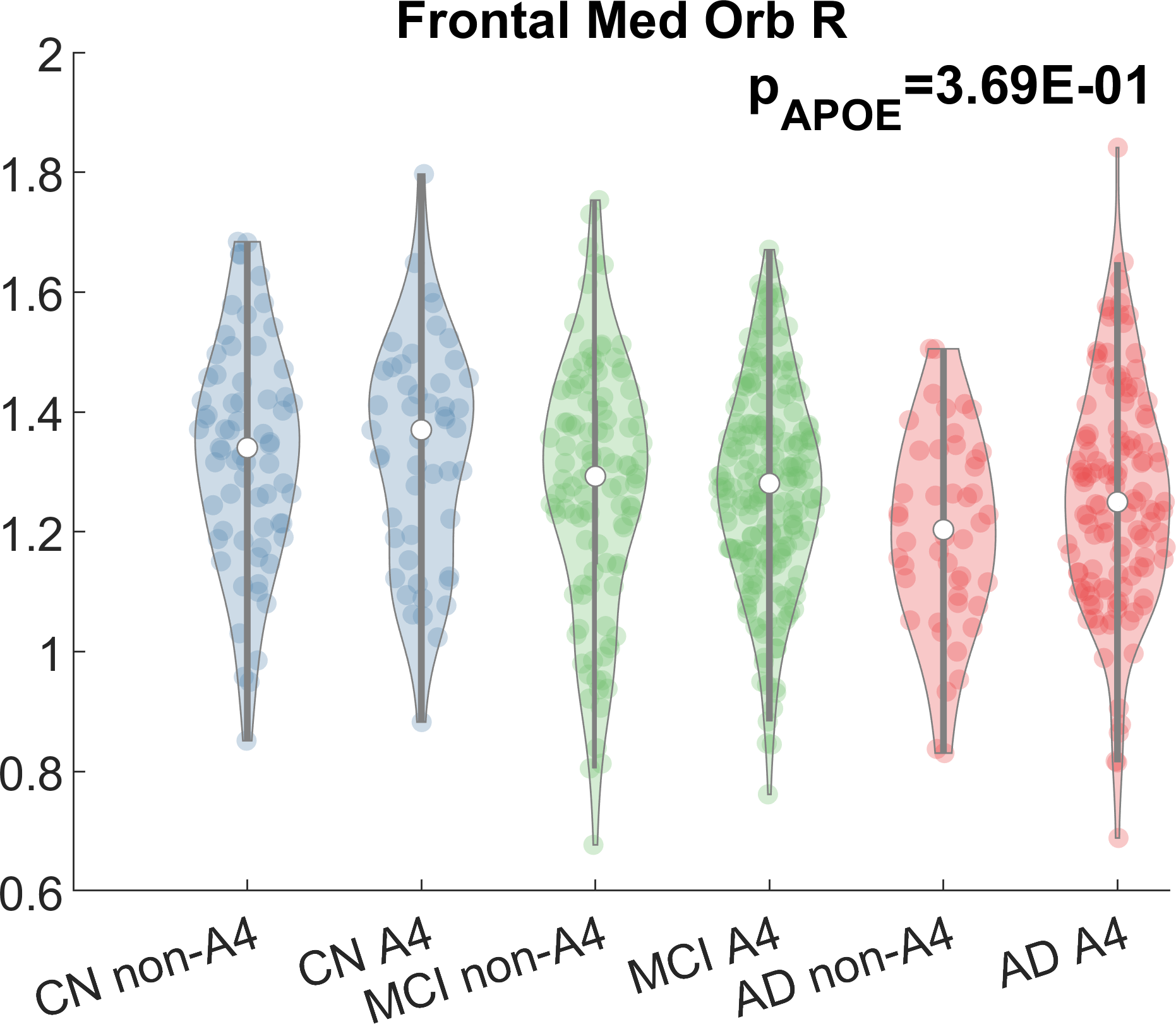

Supplement: Supplementary file 1 [file Data_Sheet_1.ZIP › Supplementary Material/Supp Figure 2/APOE4_Frontal Med Orb R.tif]

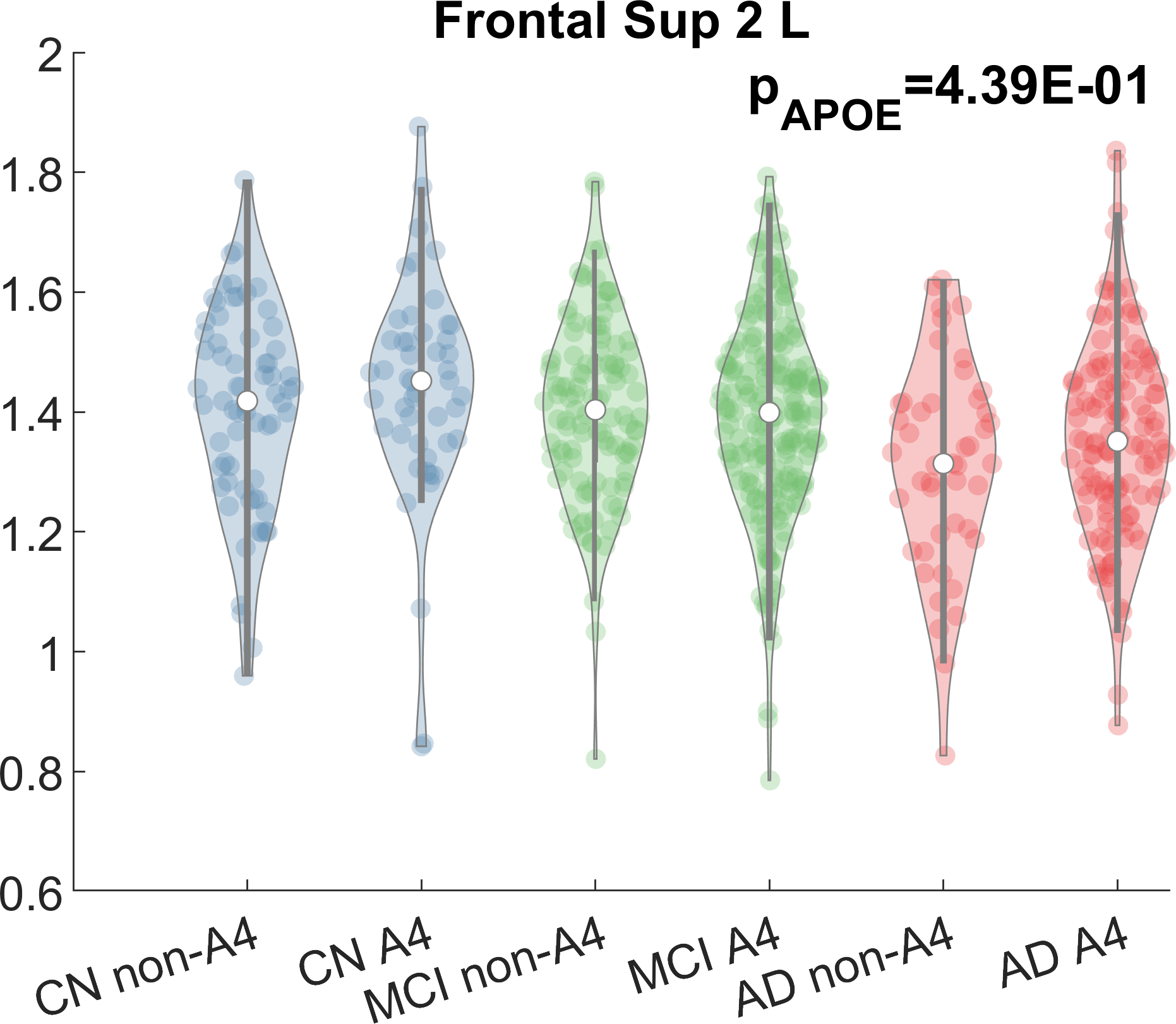

Supplement: Supplementary file 1 [file Data_Sheet_1.ZIP › Supplementary Material/Supp Figure 2/APOE4_Frontal Sup 2 L.tif]

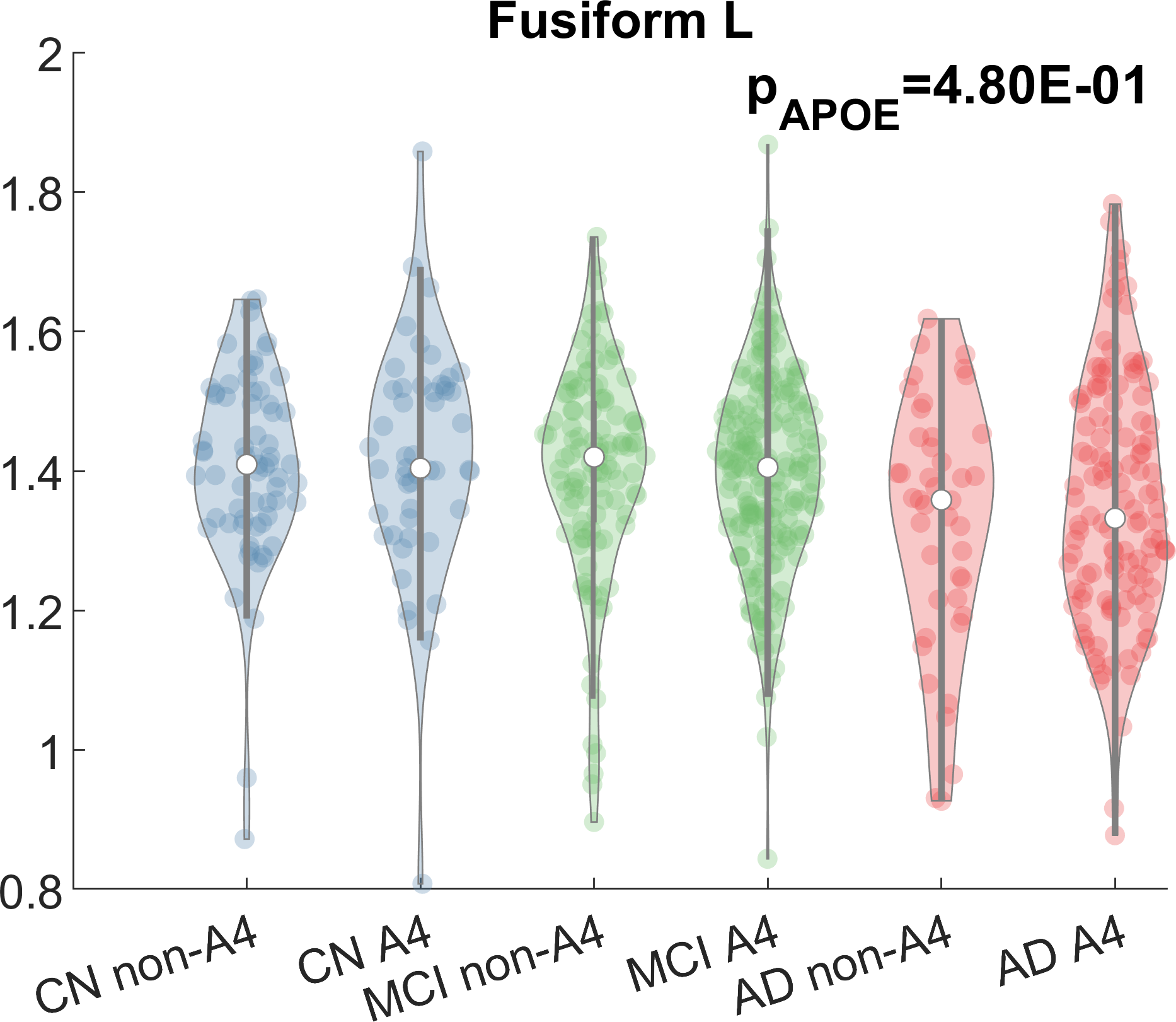

Supplement: Supplementary file 1 [file Data_Sheet_1.ZIP › Supplementary Material/Supp Figure 2/APOE4_Fusiform L.tif]

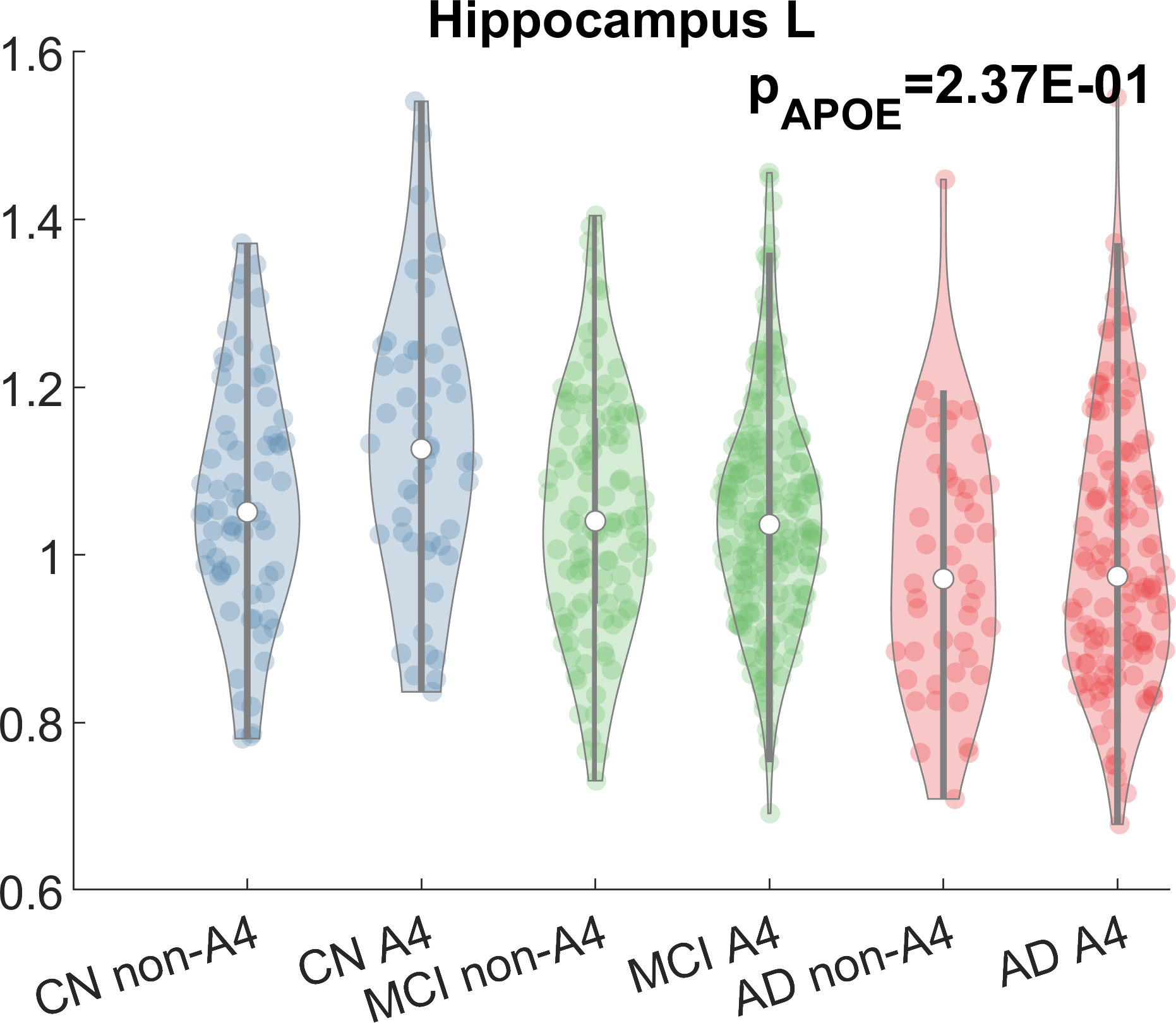

Supplement: Supplementary file 1 [file Data_Sheet_1.ZIP › Supplementary Material/Supp Figure 2/APOE4_Hippocampus L.tif]

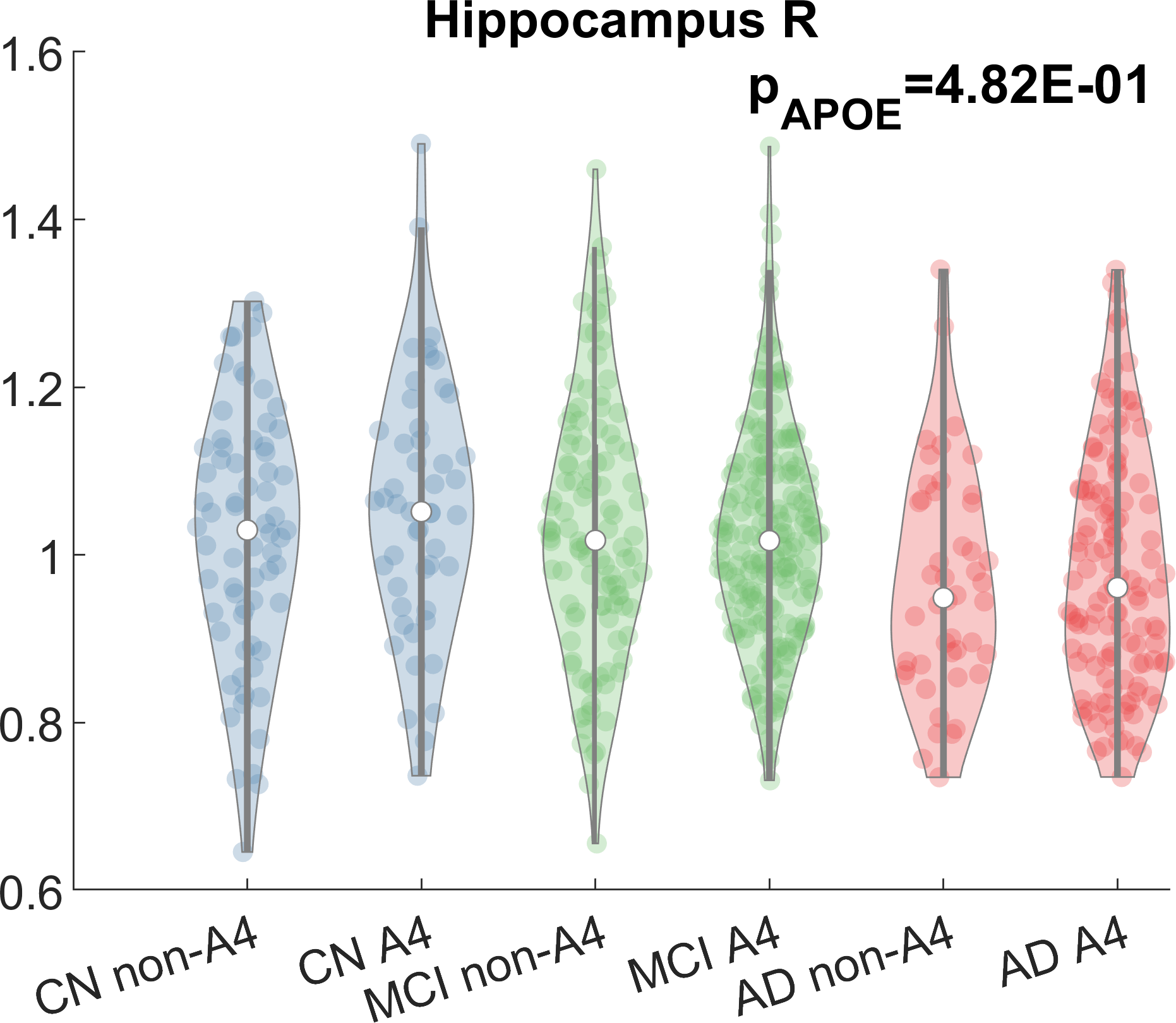

Supplement: Supplementary file 1 [file Data_Sheet_1.ZIP › Supplementary Material/Supp Figure 2/APOE4_Hippocampus R.tif]

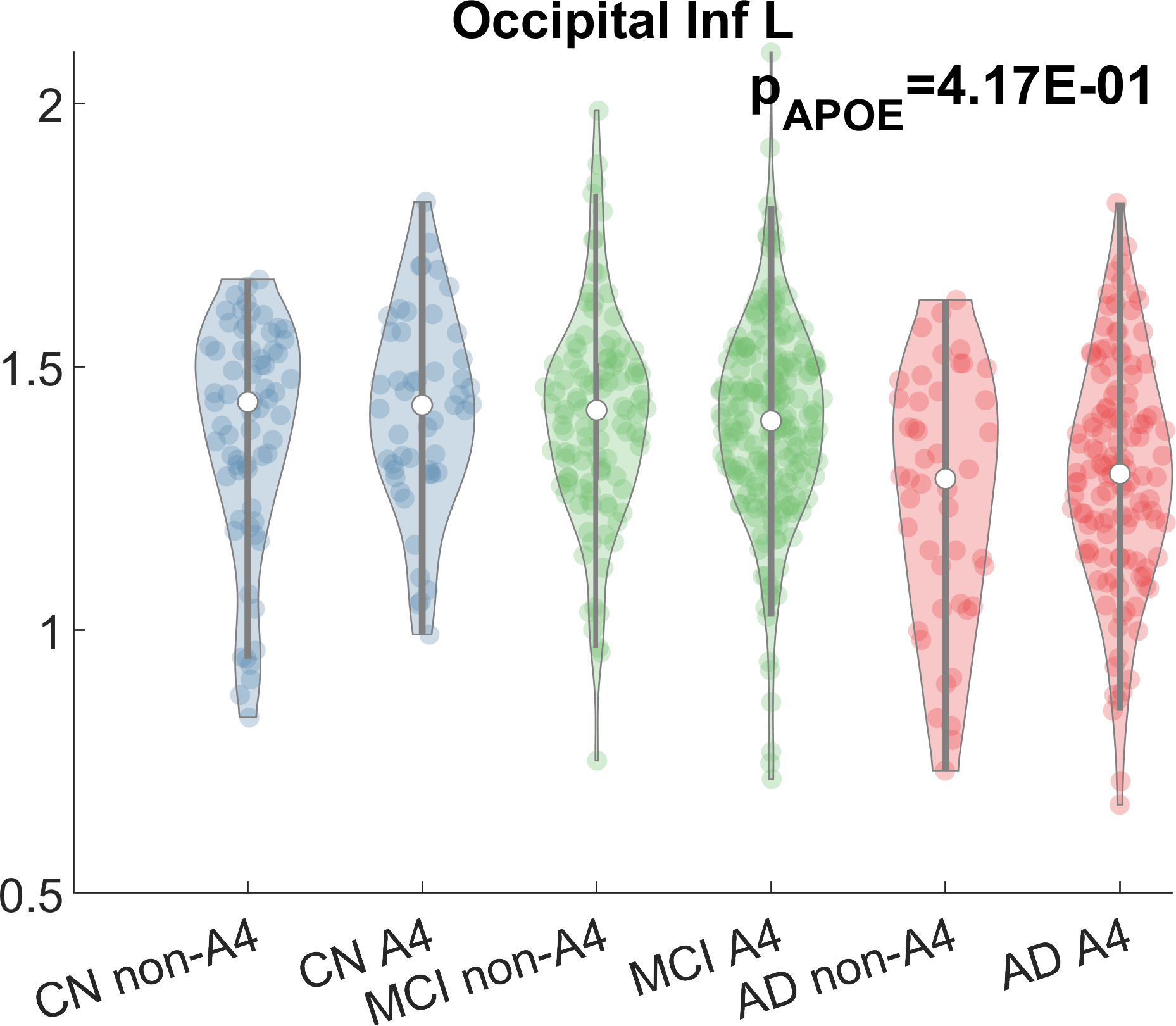

Supplement: Supplementary file 1 [file Data_Sheet_1.ZIP › Supplementary Material/Supp Figure 2/APOE4_Occipital Inf L.tif]

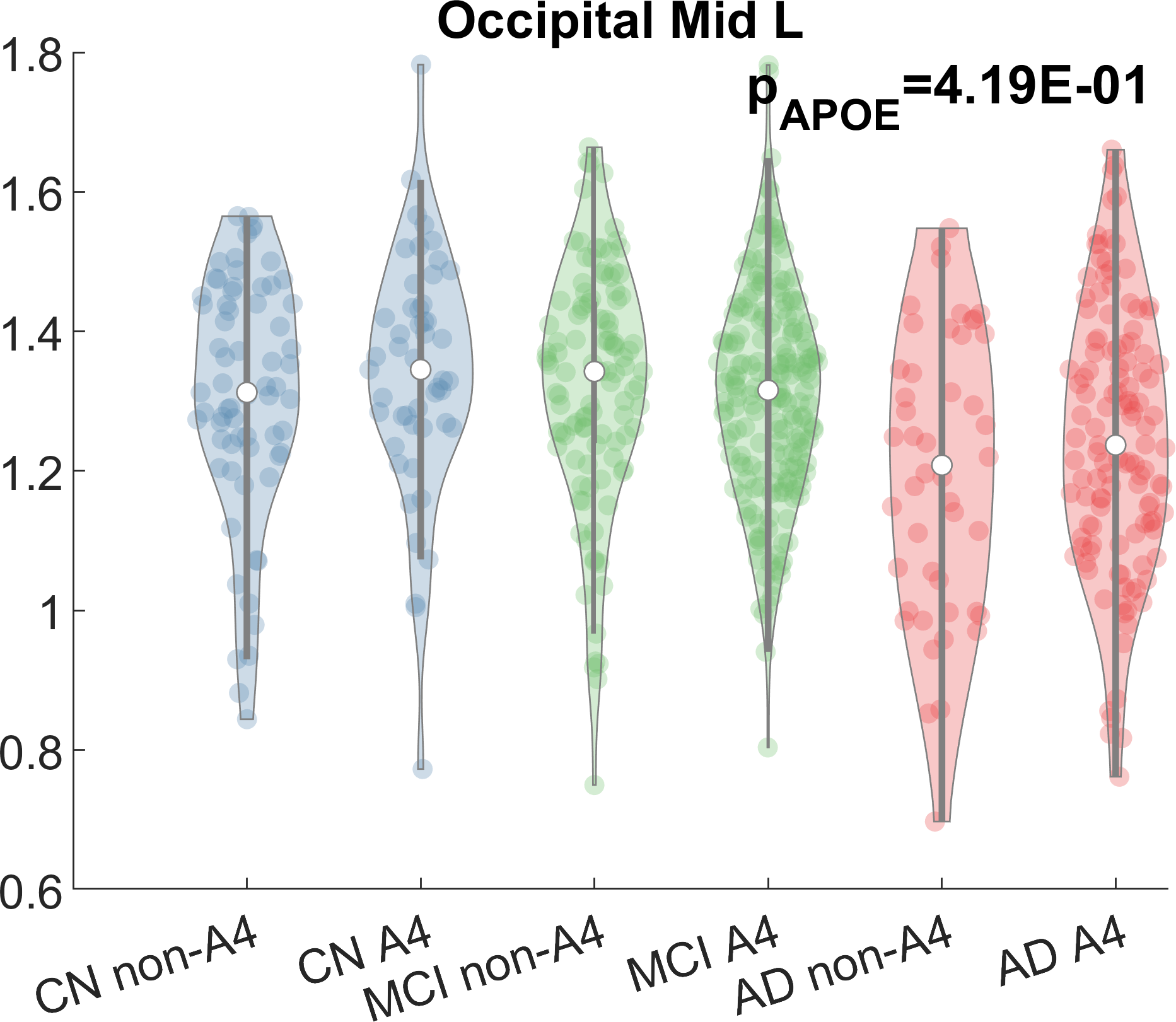

Supplement: Supplementary file 1 [file Data_Sheet_1.ZIP › Supplementary Material/Supp Figure 2/APOE4_Occipital Mid L.tif]

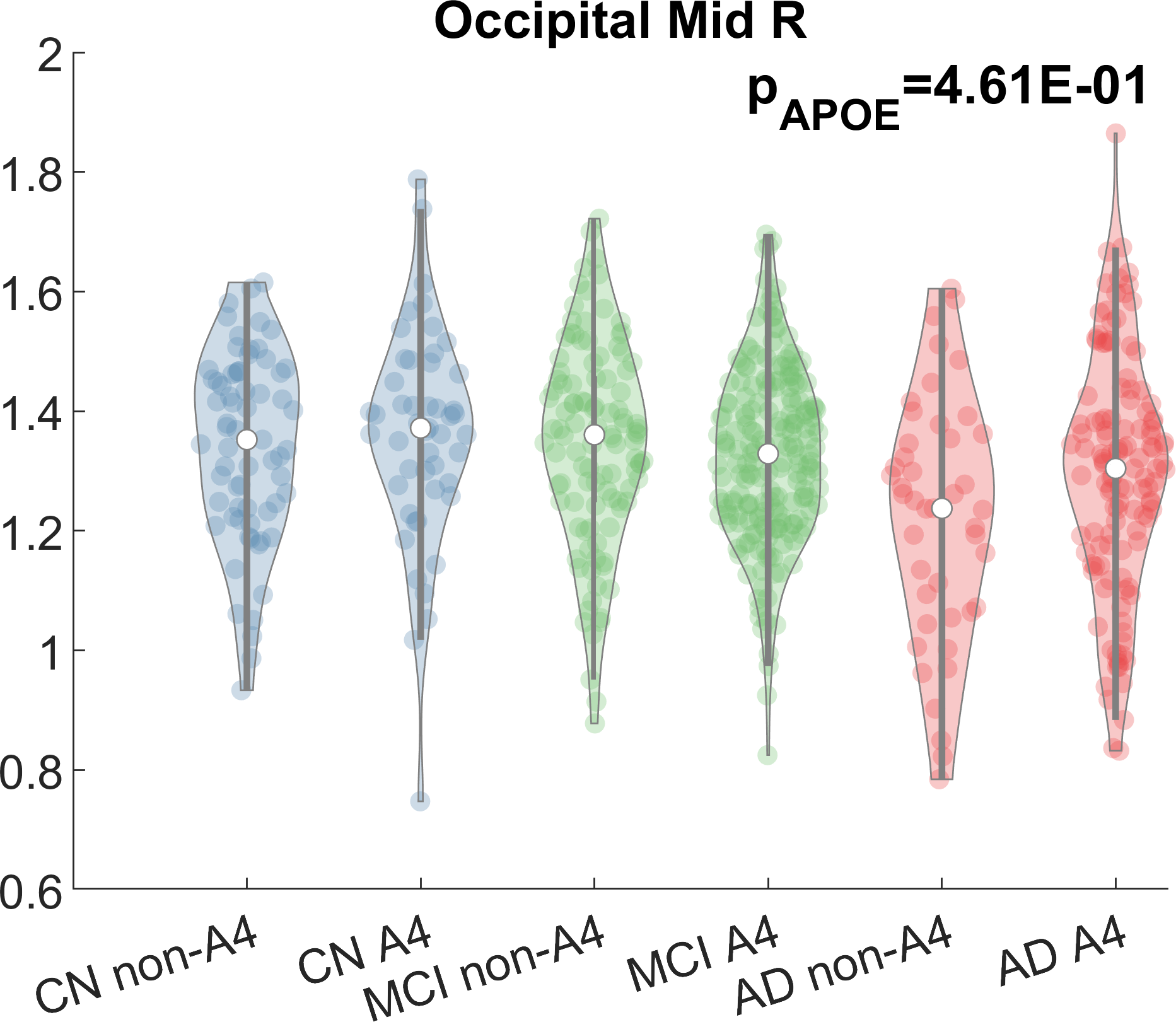

Supplement: Supplementary file 1 [file Data_Sheet_1.ZIP › Supplementary Material/Supp Figure 2/APOE4_Occipital Mid R.tif]

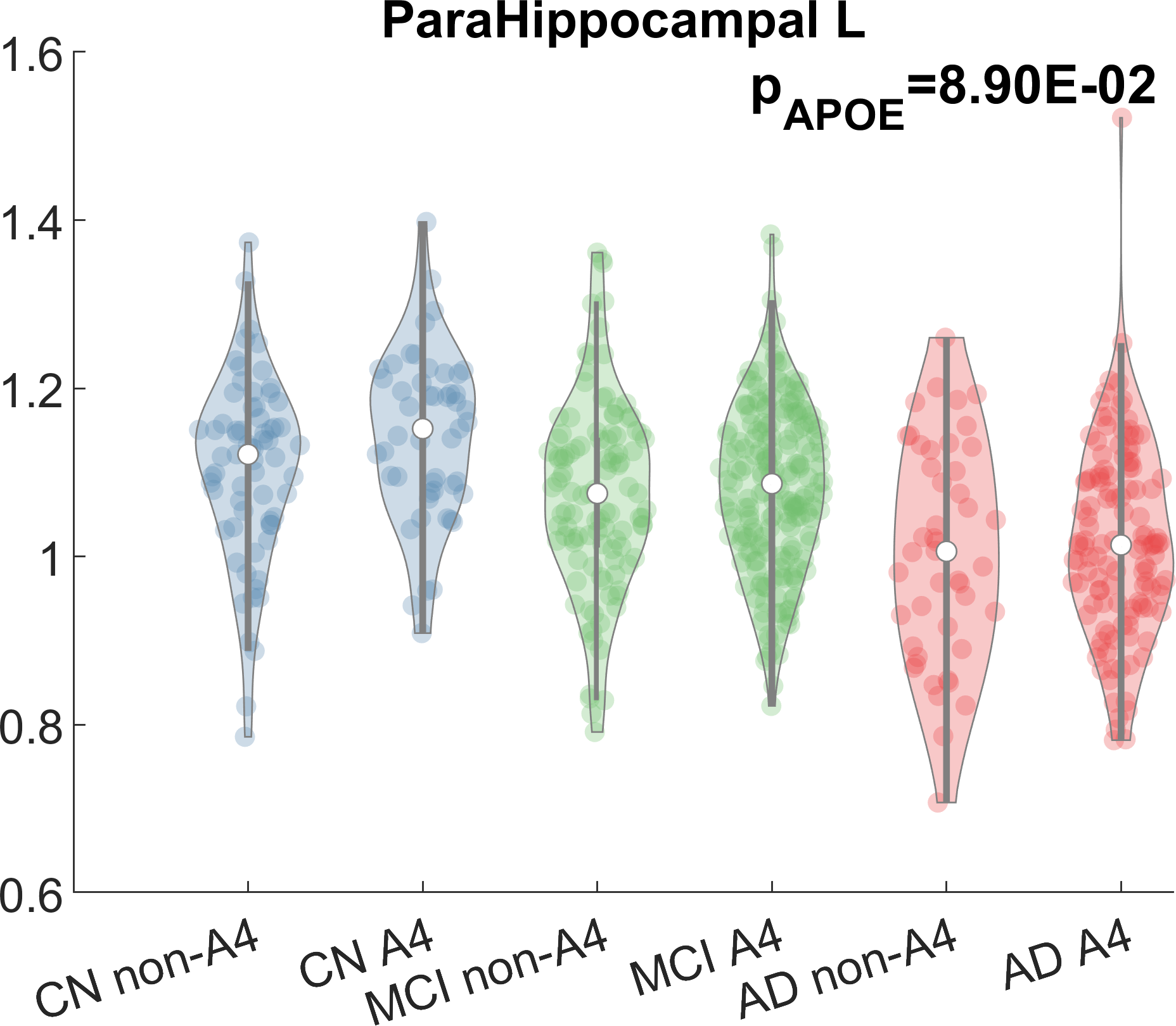

Supplement: Supplementary file 1 [file Data_Sheet_1.ZIP › Supplementary Material/Supp Figure 2/APOE4_ParaHippocampal L.tif]

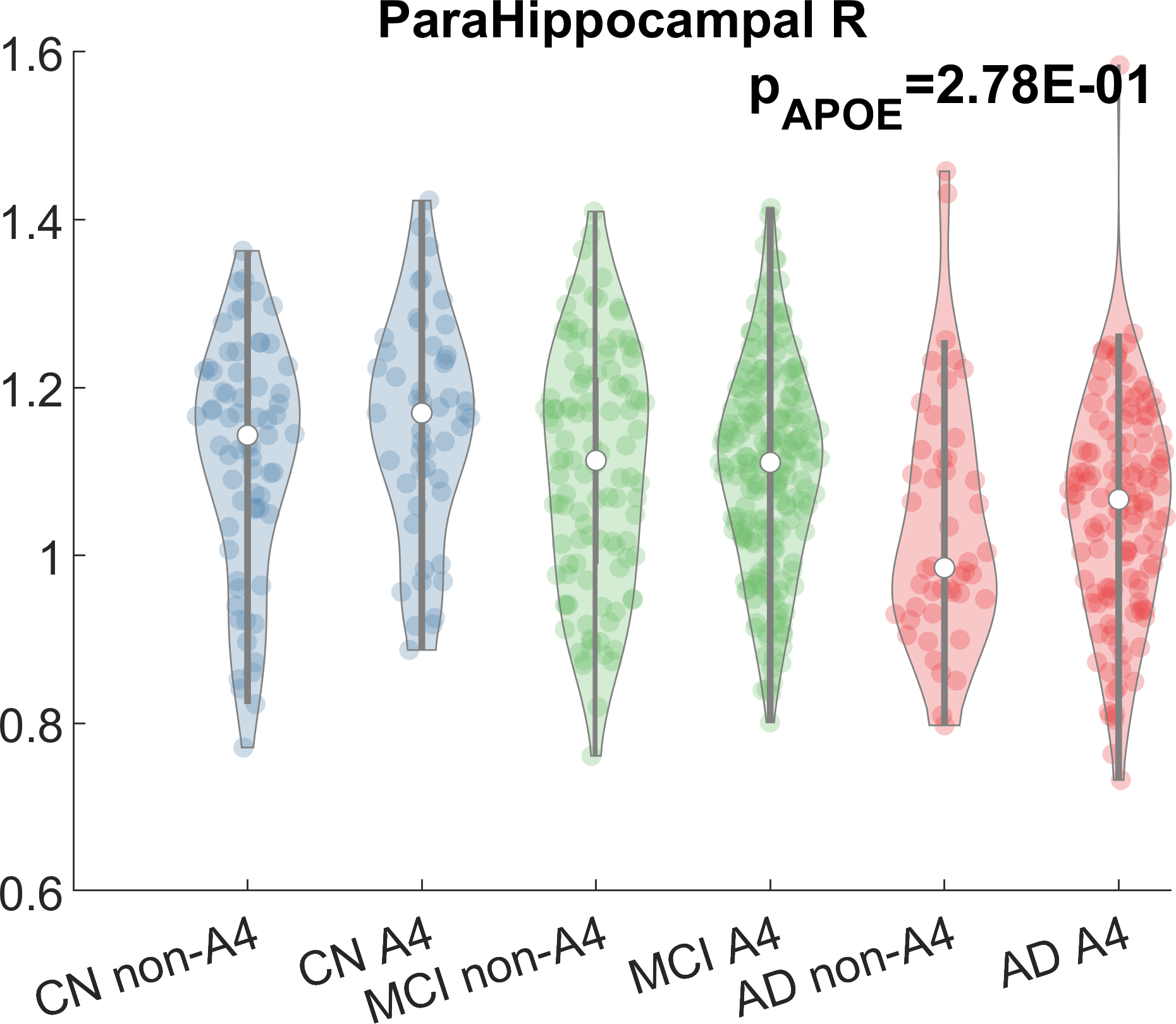

Supplement: Supplementary file 1 [file Data_Sheet_1.ZIP › Supplementary Material/Supp Figure 2/APOE4_ParaHippocampal R.tif]

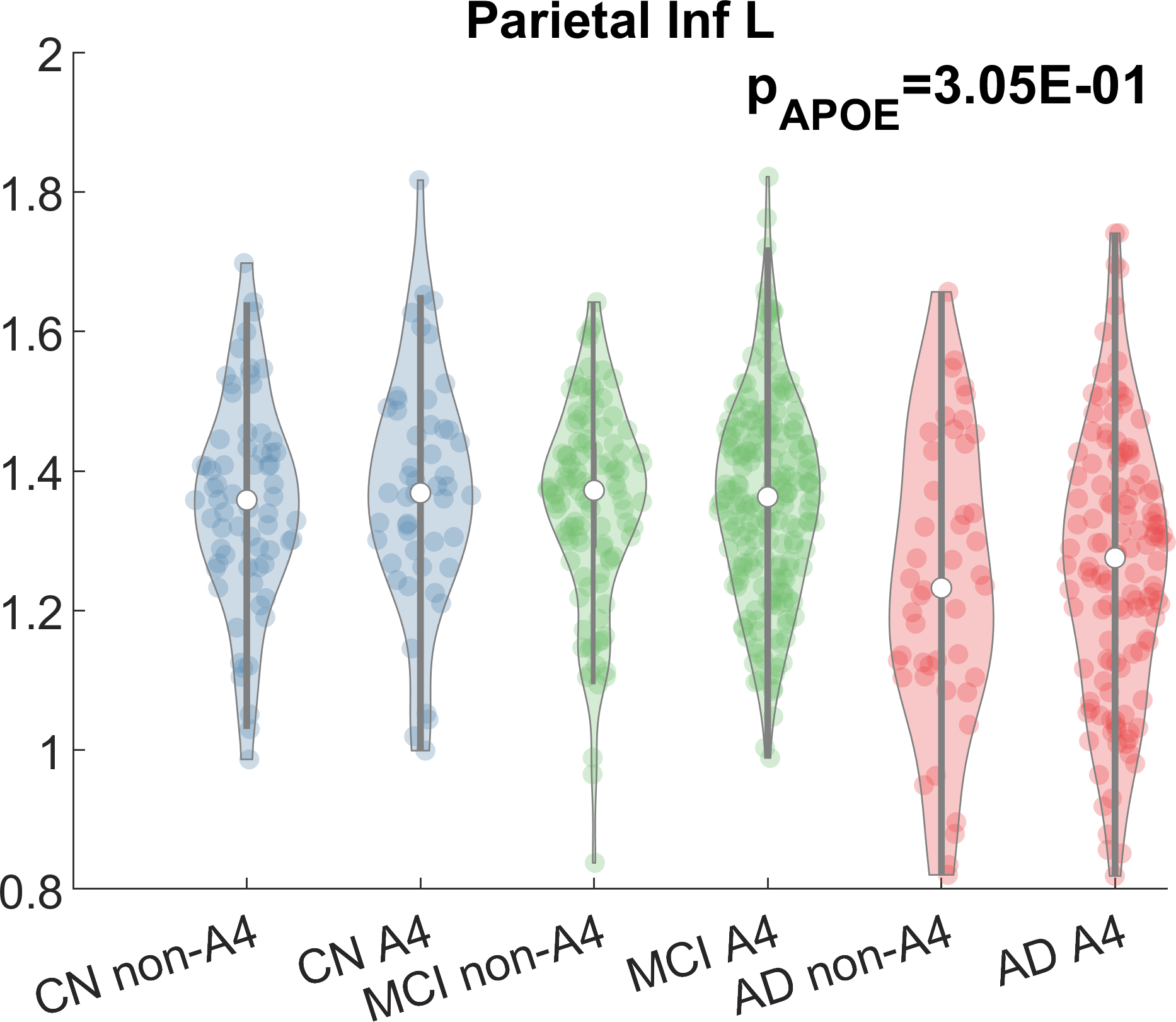

Supplement: Supplementary file 1 [file Data_Sheet_1.ZIP › Supplementary Material/Supp Figure 2/APOE4_Parietal Inf L.tif]

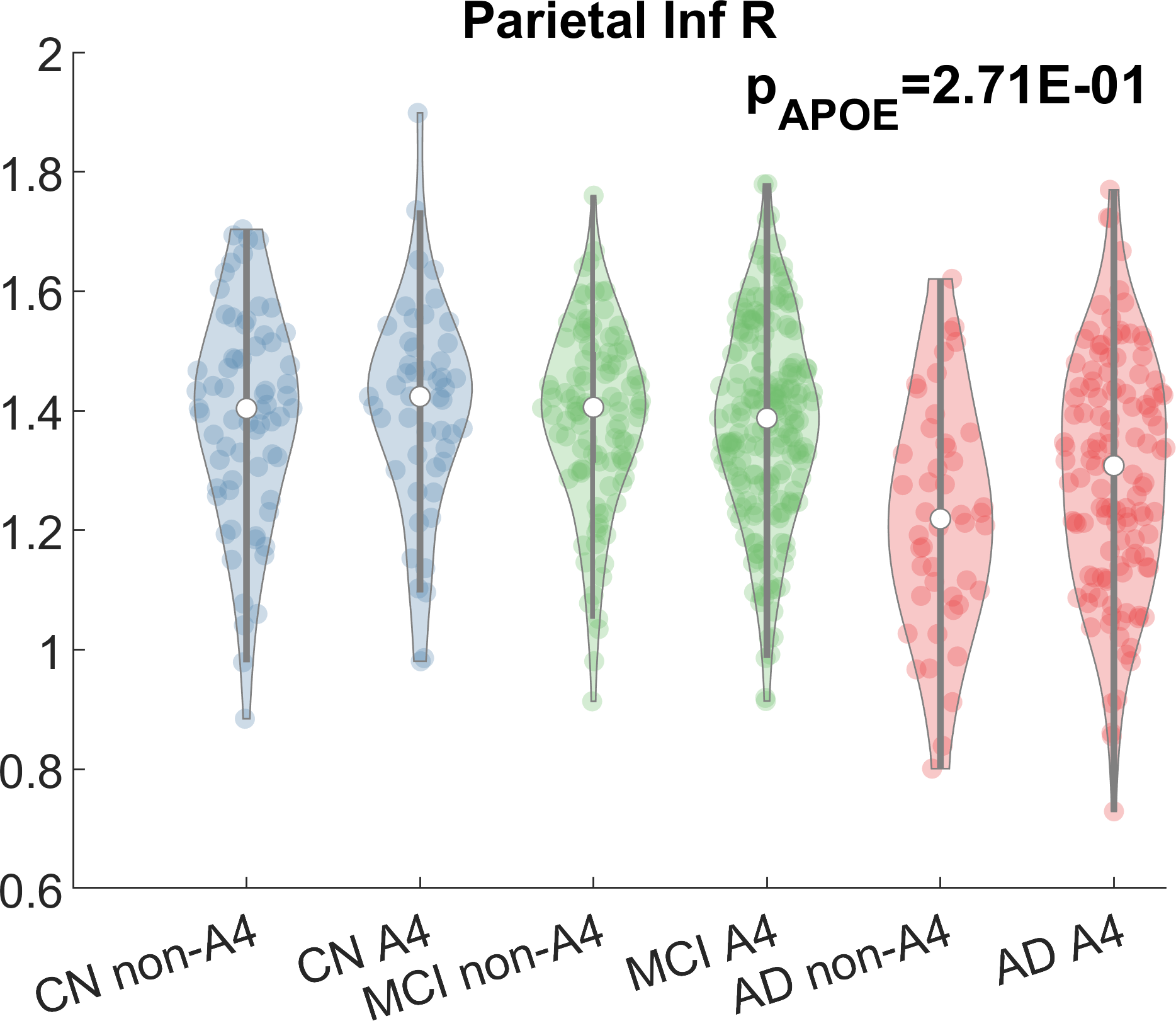

Supplement: Supplementary file 1 [file Data_Sheet_1.ZIP › Supplementary Material/Supp Figure 2/APOE4_Parietal Inf R.tif]

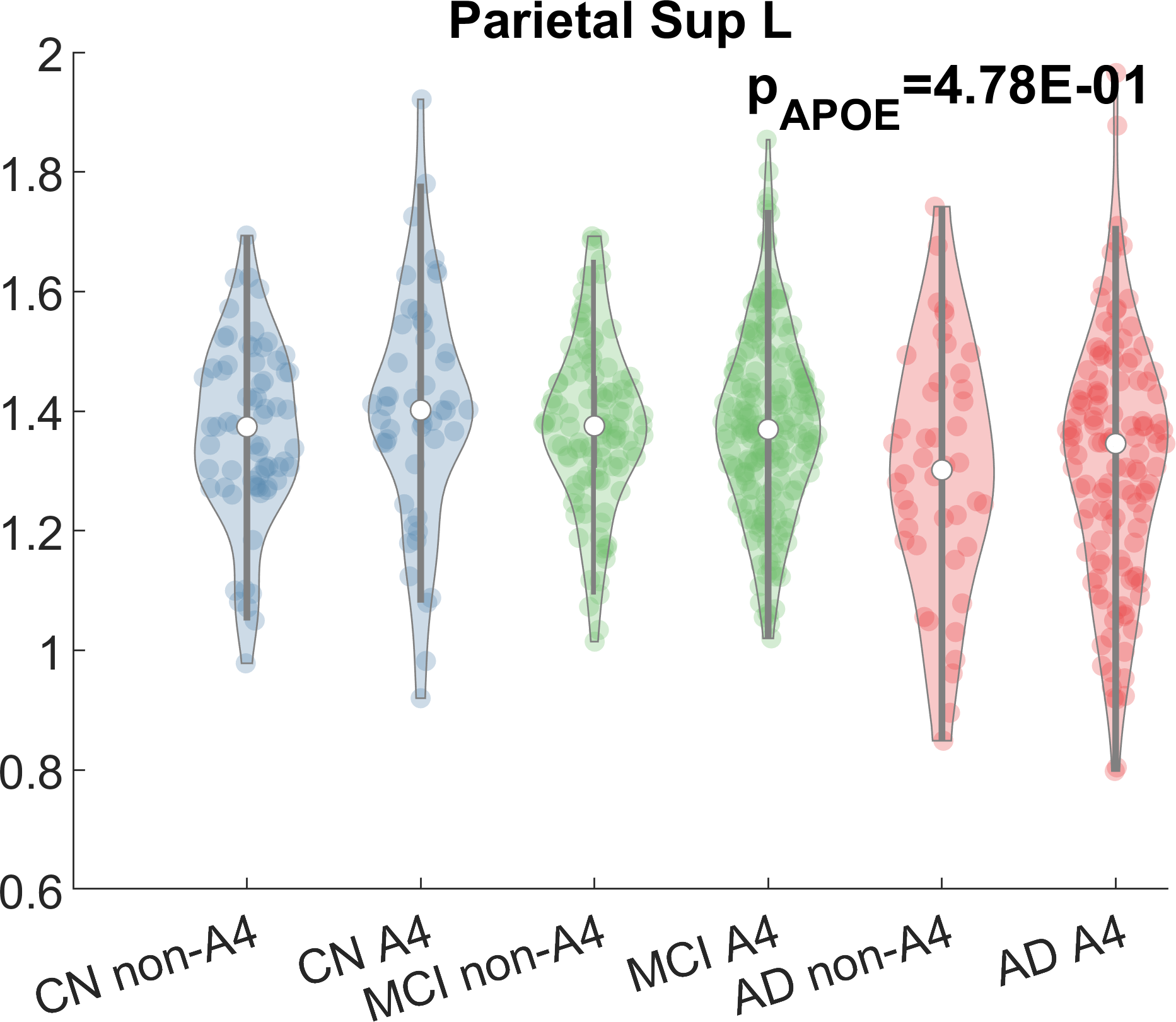

Supplement: Supplementary file 1 [file Data_Sheet_1.ZIP › Supplementary Material/Supp Figure 2/APOE4_Parietal Sup L.tif]

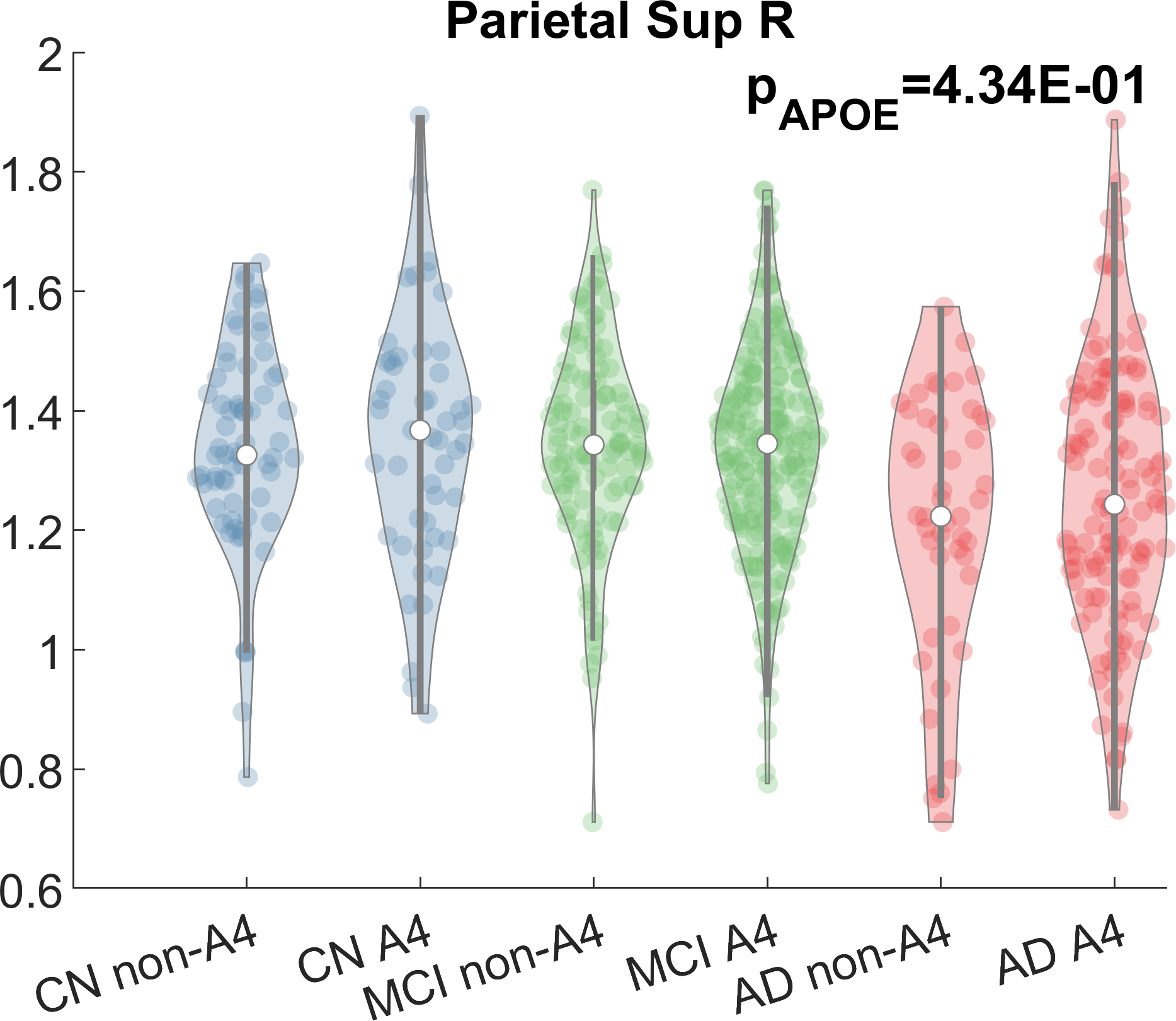

Supplement: Supplementary file 1 [file Data_Sheet_1.ZIP › Supplementary Material/Supp Figure 2/APOE4_Parietal Sup R.tif]

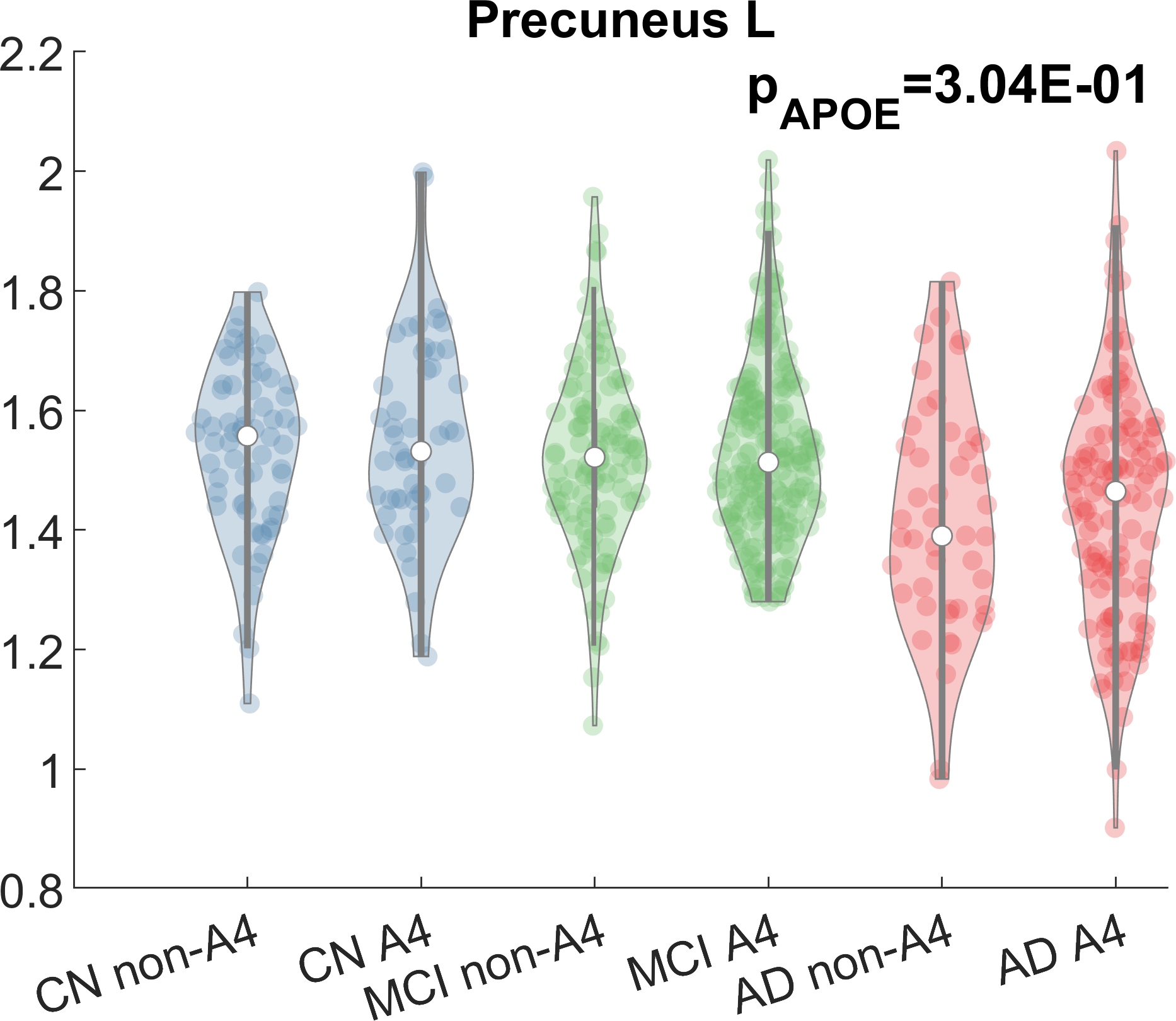

Supplement: Supplementary file 1 [file Data_Sheet_1.ZIP › Supplementary Material/Supp Figure 2/APOE4_Precuneus L.tif]

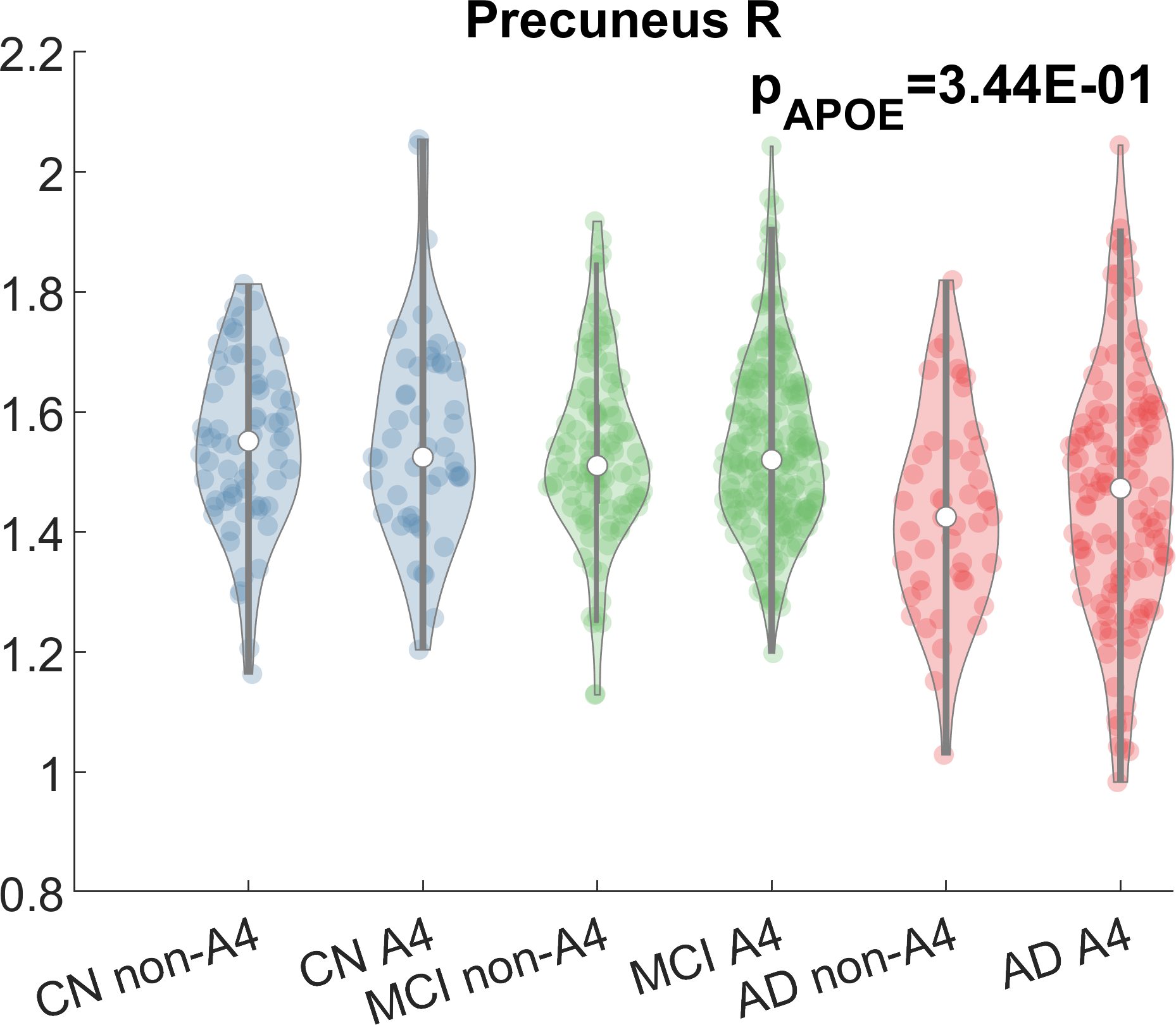

Supplement: Supplementary file 1 [file Data_Sheet_1.ZIP › Supplementary Material/Supp Figure 2/APOE4_Precuneus R.tif]

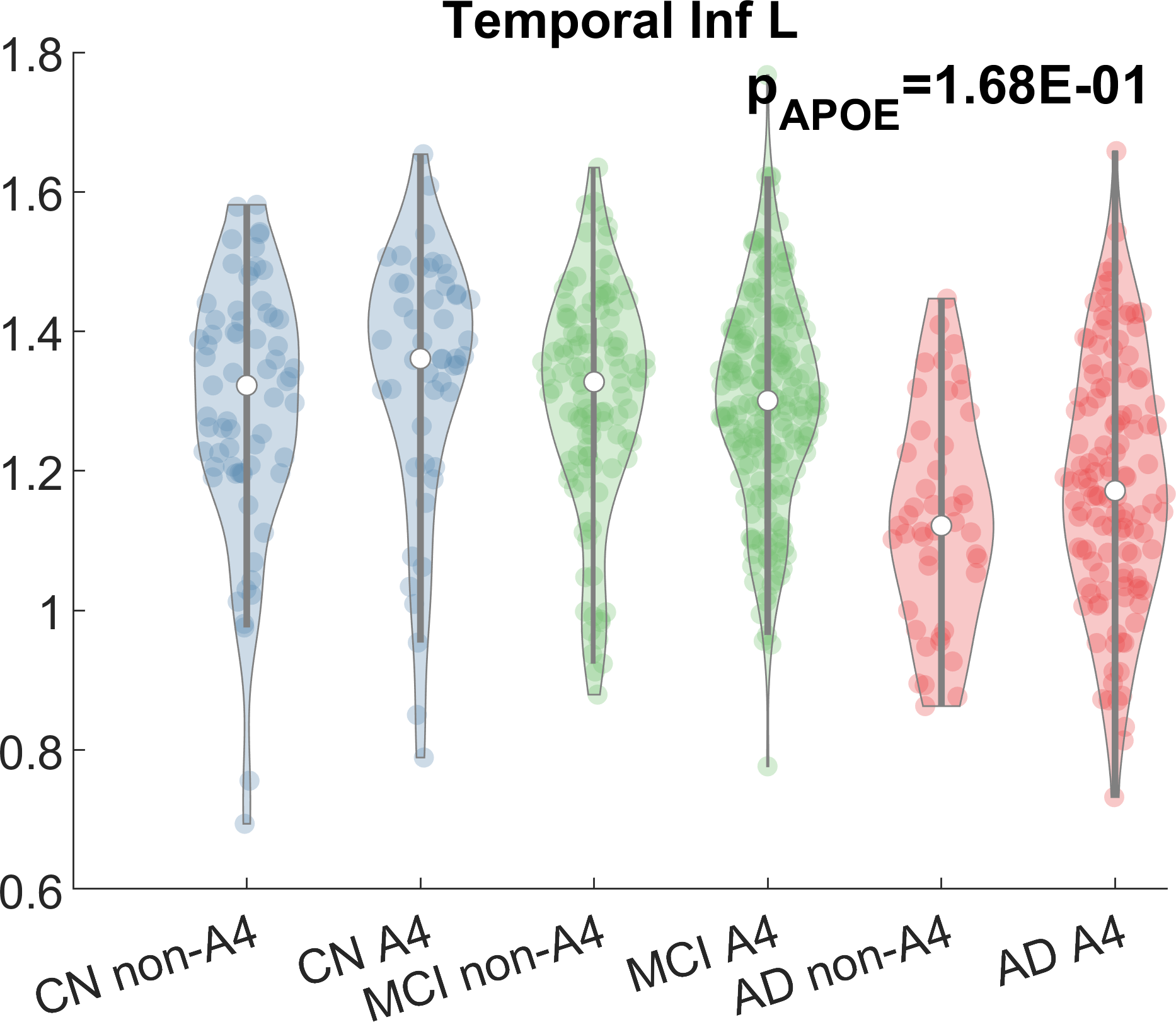

Supplement: Supplementary file 1 [file Data_Sheet_1.ZIP › Supplementary Material/Supp Figure 2/APOE4_Temporal Inf L.tif]

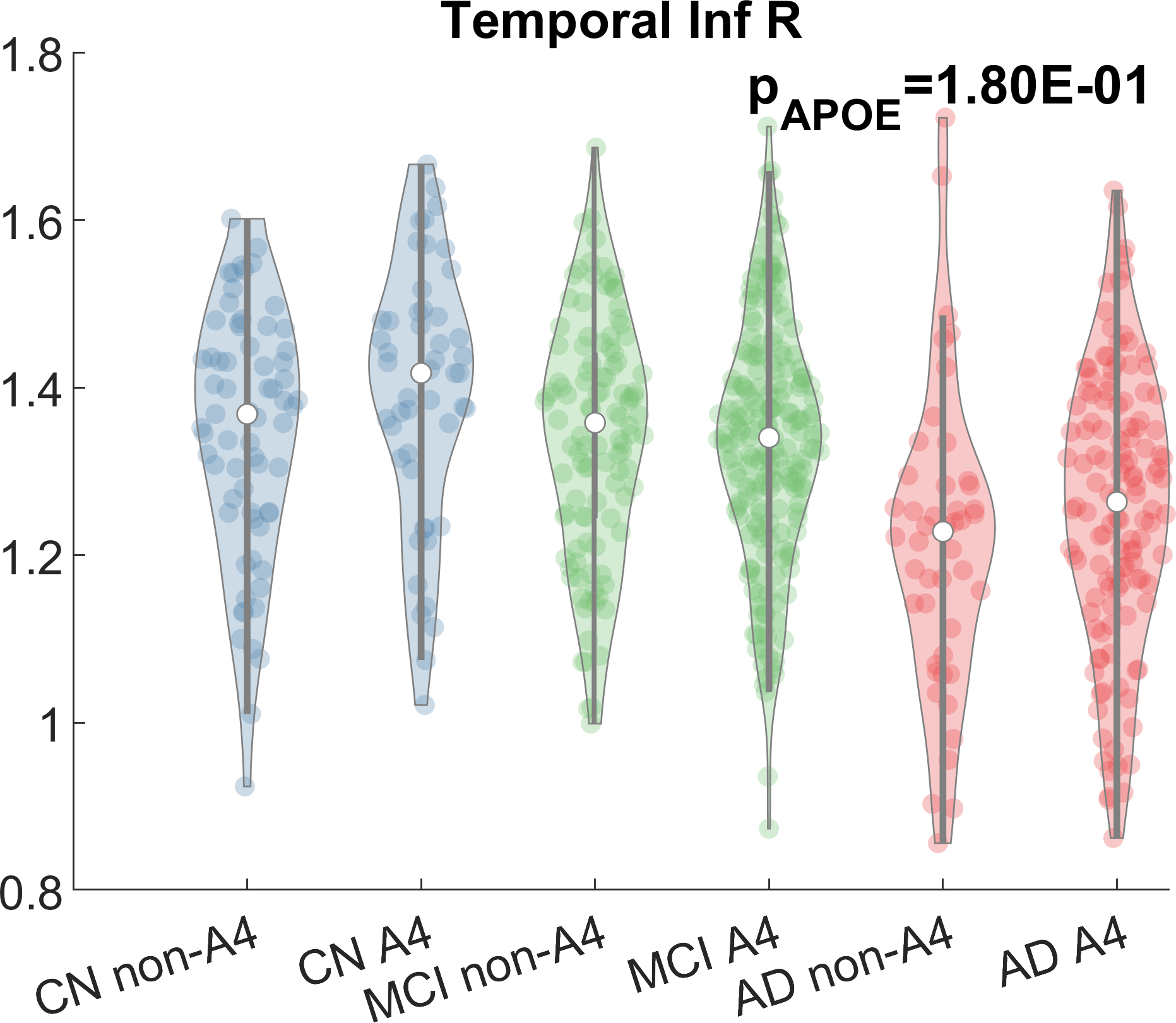

Supplement: Supplementary file 1 [file Data_Sheet_1.ZIP › Supplementary Material/Supp Figure 2/APOE4_Temporal Inf R.tif]

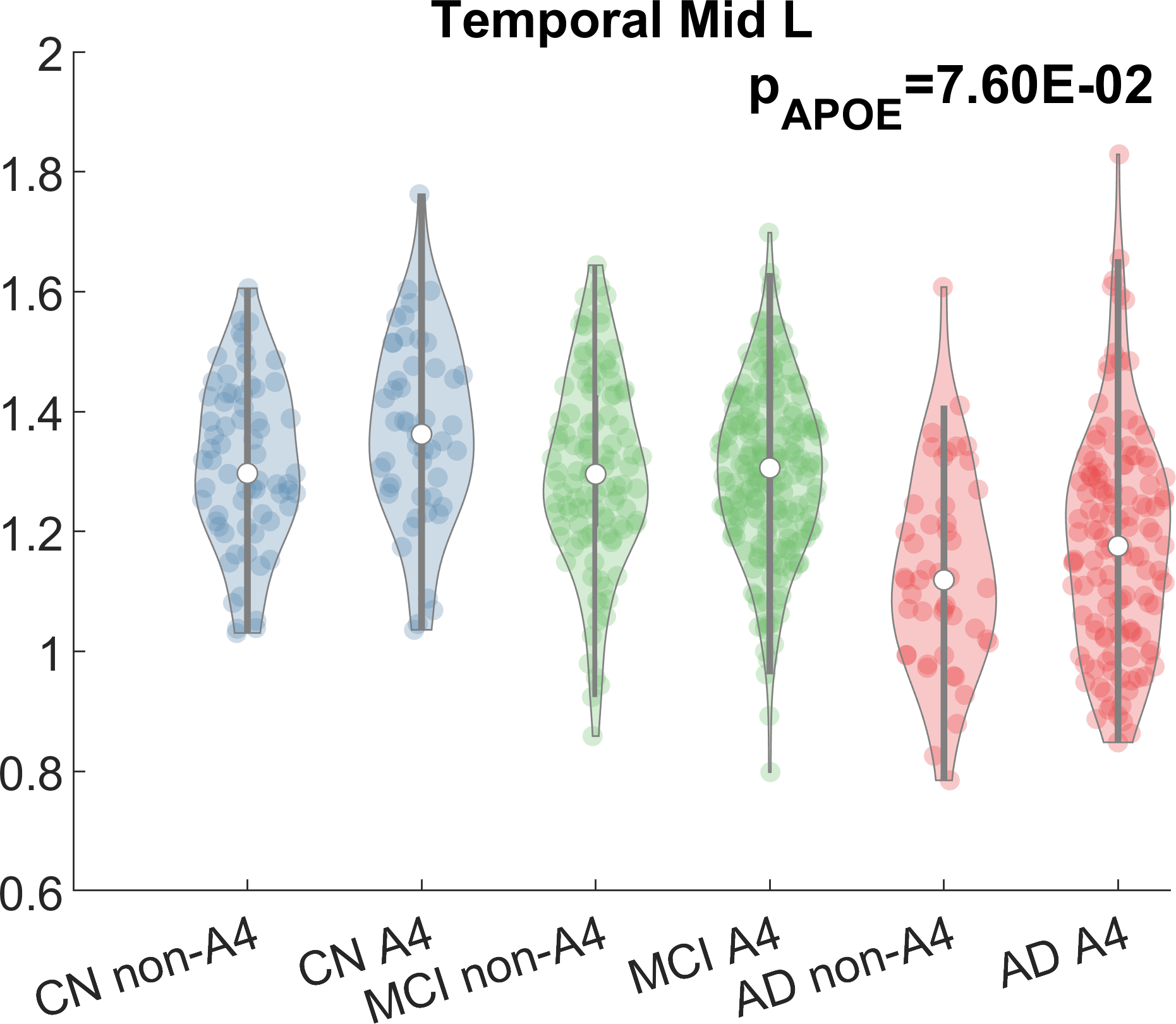

Supplement: Supplementary file 1 [file Data_Sheet_1.ZIP › Supplementary Material/Supp Figure 2/APOE4_Temporal Mid L.tif]

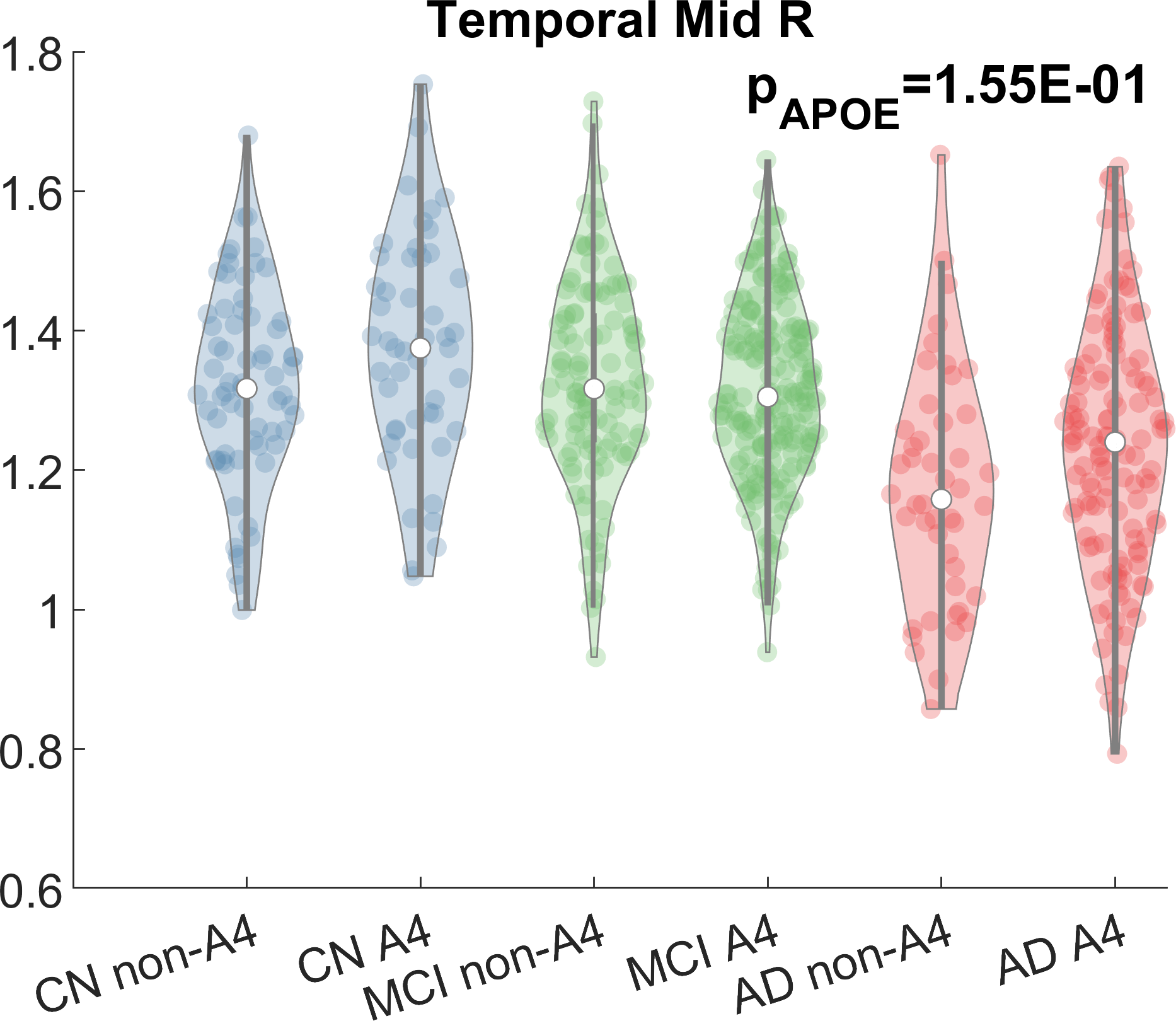

Supplement: Supplementary file 1 [file Data_Sheet_1.ZIP › Supplementary Material/Supp Figure 2/APOE4_Temporal Mid R.tif]

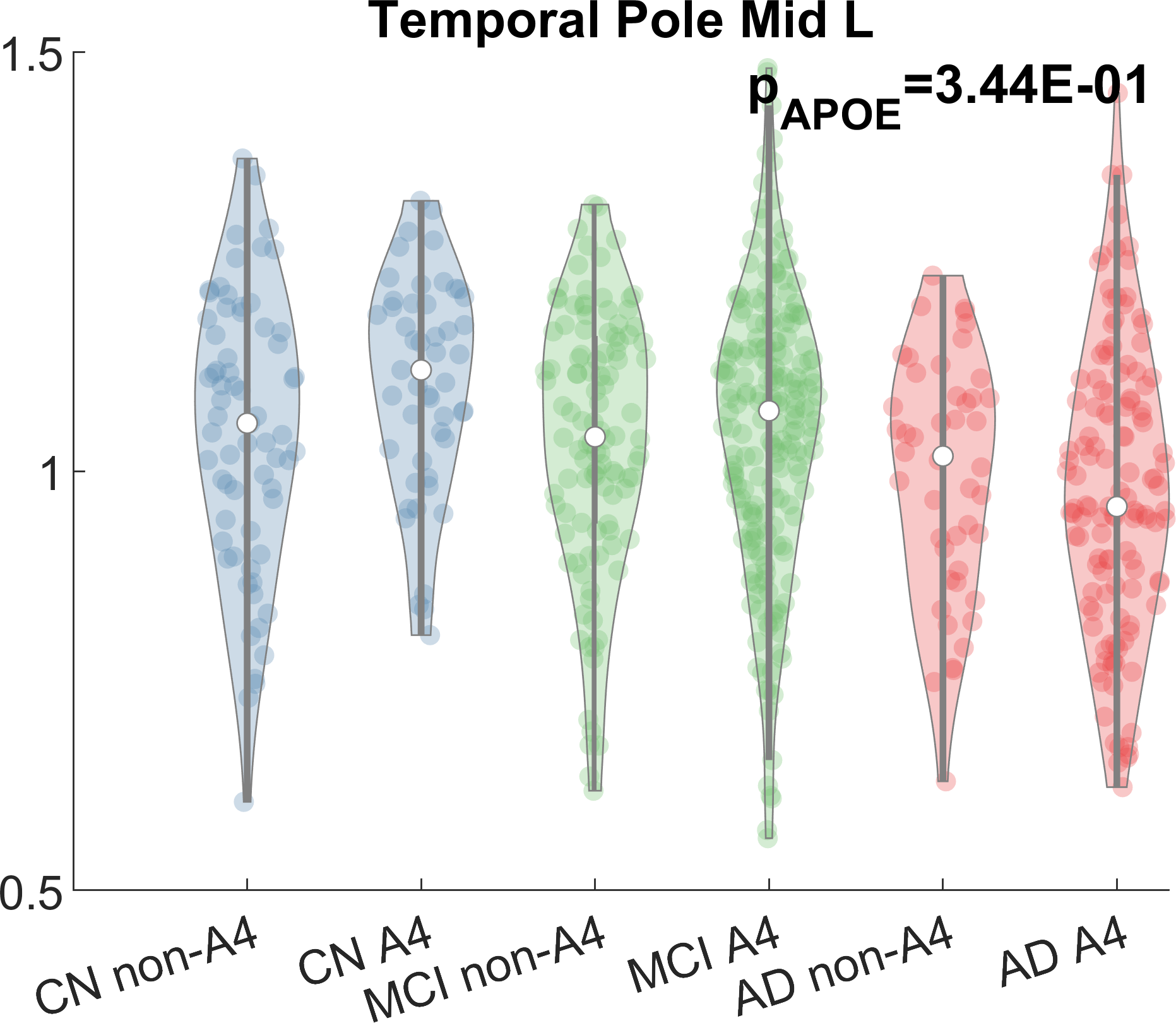

Supplement: Supplementary file 1 [file Data_Sheet_1.ZIP › Supplementary Material/Supp Figure 2/APOE4_Temporal Pole Mid L.tif]

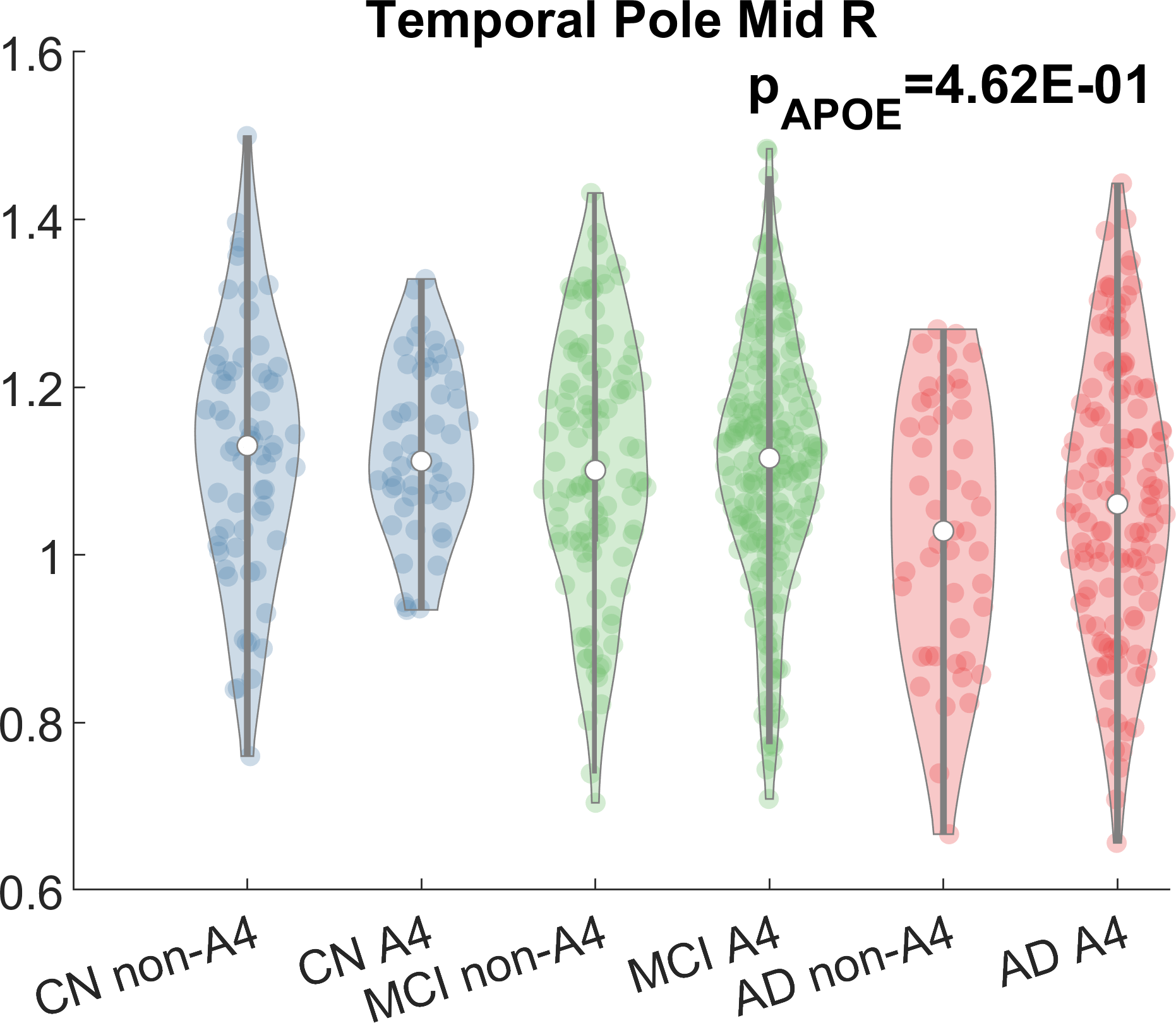

Supplement: Supplementary file 1 [file Data_Sheet_1.ZIP › Supplementary Material/Supp Figure 2/APOE4_Temporal Pole Mid R.tif]

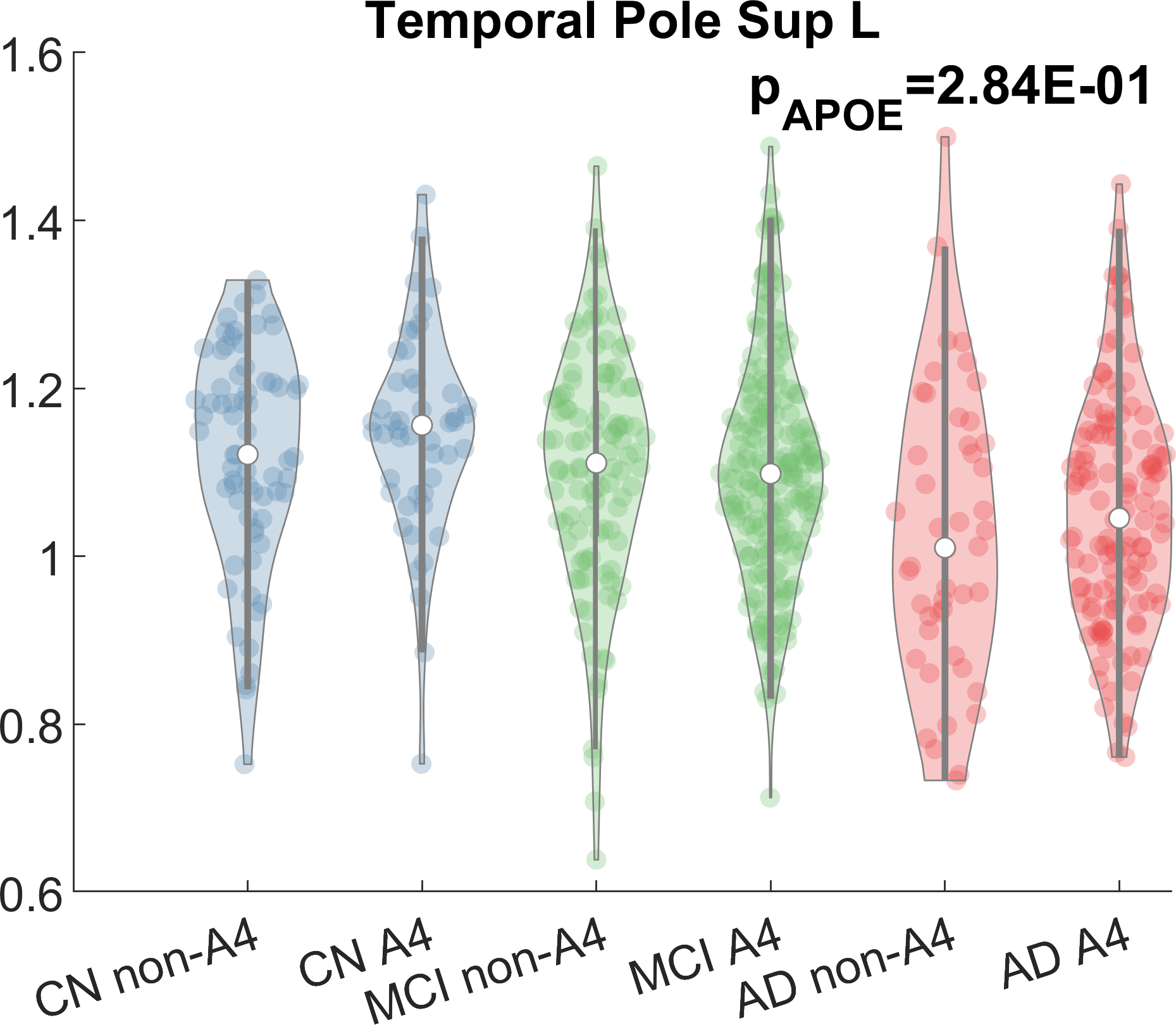

Supplement: Supplementary file 1 [file Data_Sheet_1.ZIP › Supplementary Material/Supp Figure 2/APOE4_Temporal Pole Sup L.tif]

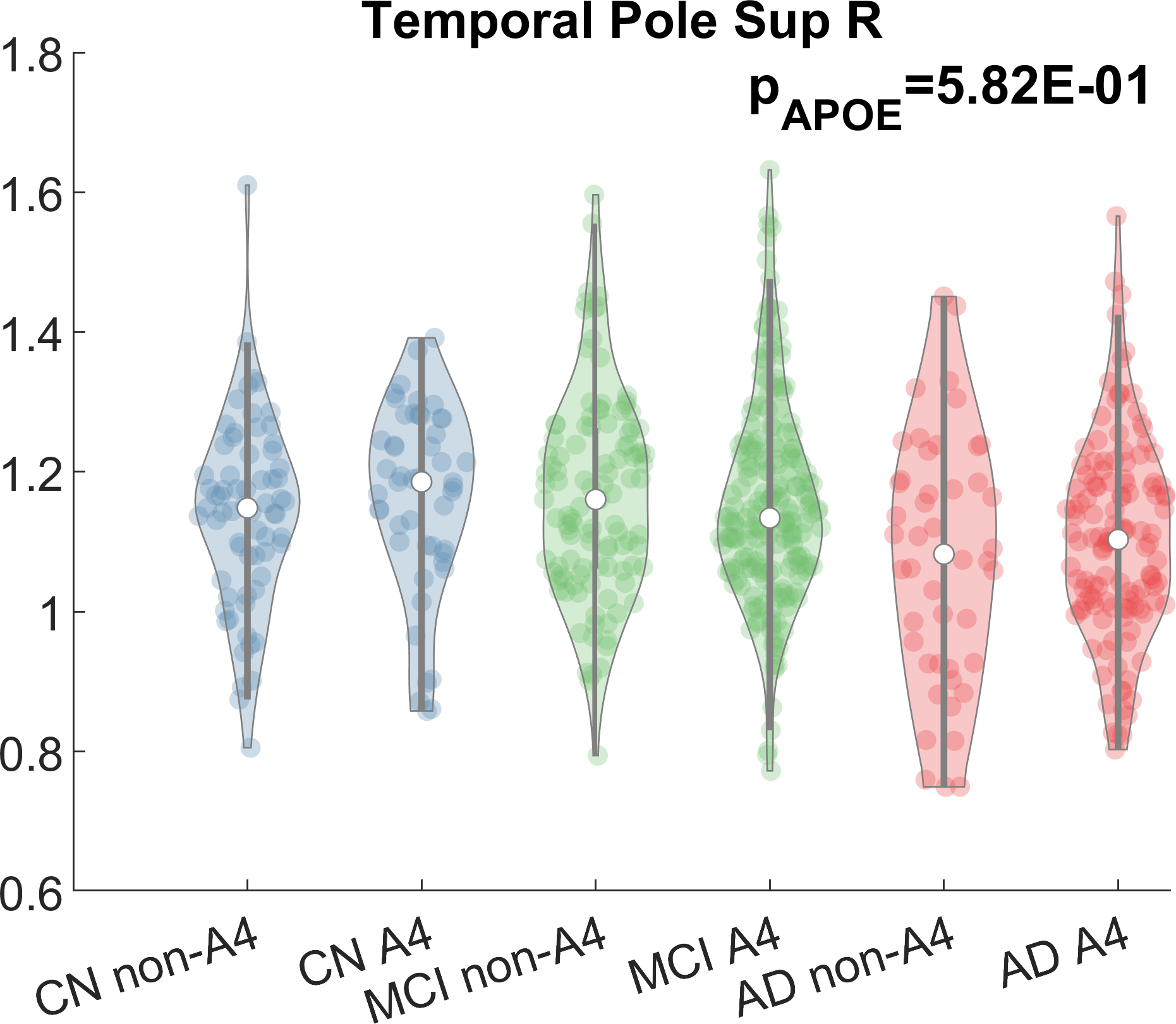

Supplement: Supplementary file 1 [file Data_Sheet_1.ZIP › Supplementary Material/Supp Figure 2/APOE4_Temporal Pole Sup R.tif]

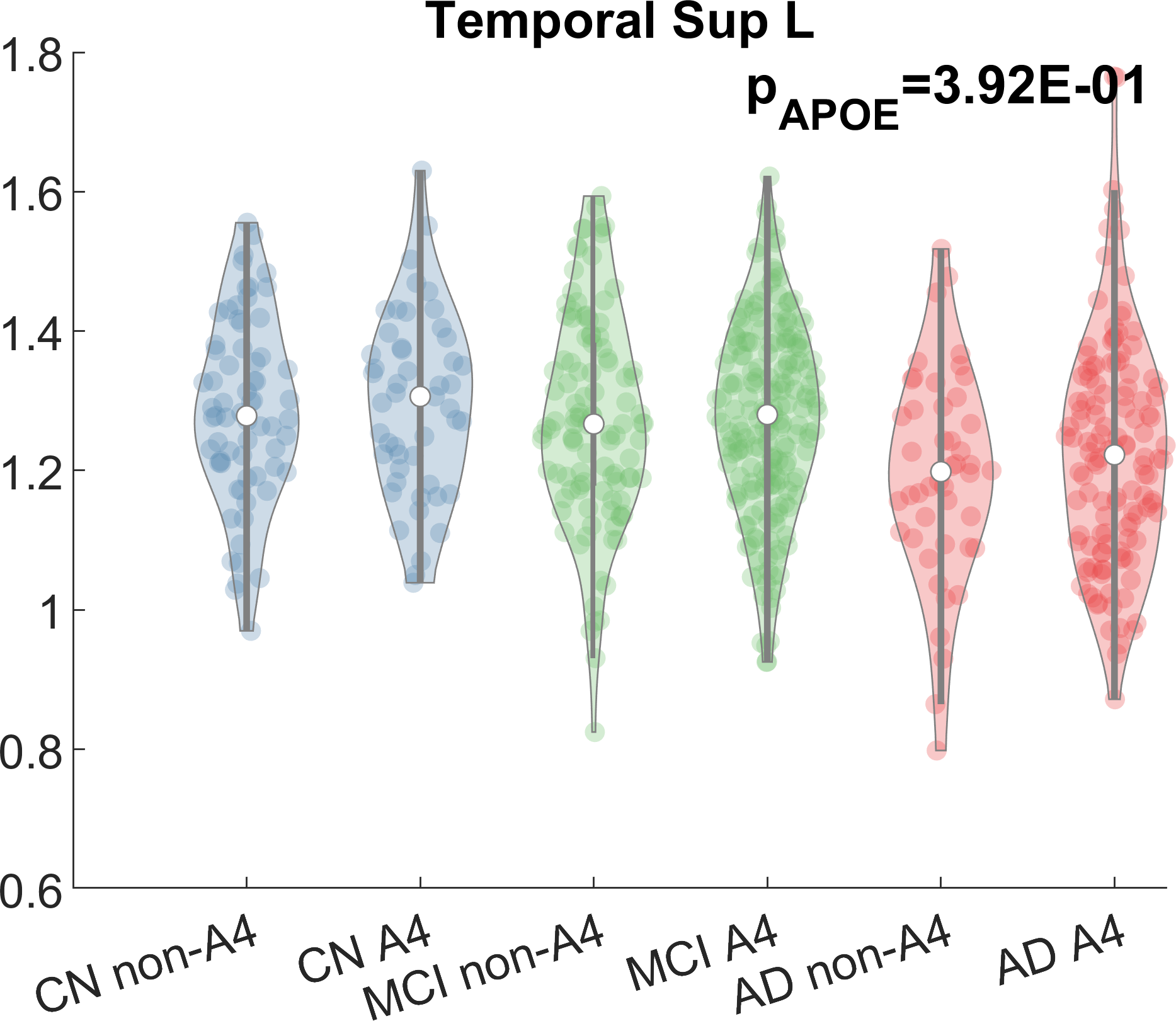

Supplement: Supplementary file 1 [file Data_Sheet_1.ZIP › Supplementary Material/Supp Figure 2/APOE4_Temporal Sup L.tif]

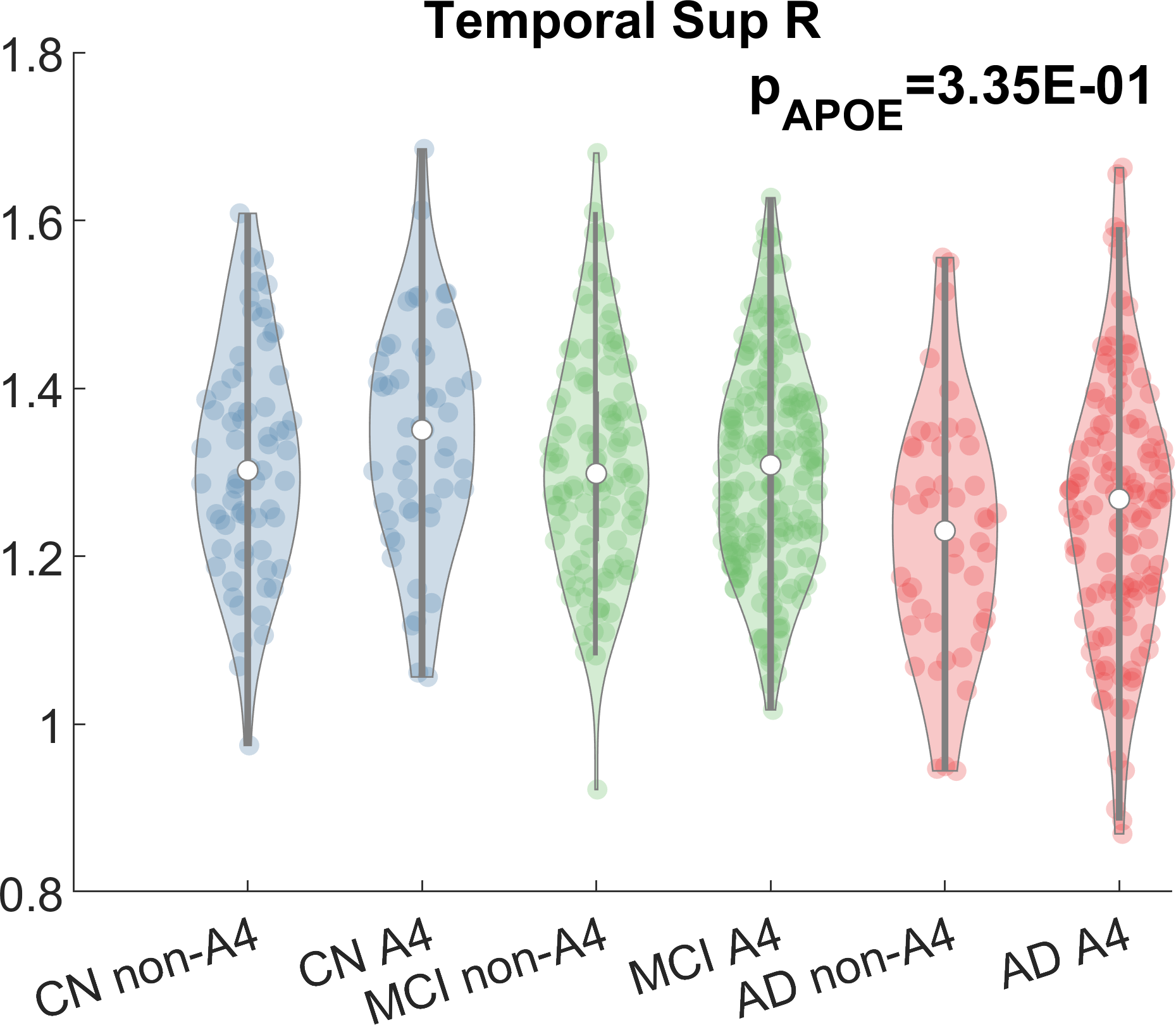

Supplement: Supplementary file 1 [file Data_Sheet_1.ZIP › Supplementary Material/Supp Figure 2/APOE4_Temporal Sup R.tif]
